# Supplementary material for: Reexamining the Kuleshov effect: Behavioral and neural evidence from authentic film experiments
Source: PLoS One. 2024 Aug 5;19(8):e0308295. doi: 10.1371/journal.pone.0308295 (PMC11299807; doi:10.1371/journal.pone.0308295)
Supplement: S1 Text — The detailed methods of materials rating experiment. (DOCX) [file pone.0308295.s001.docx]

**Reexamining the Kuleshov effect: behavioral and neural evidence from authentic film experiments**

*Materials Rating Experiment*

**Supplementary methods**

1.1. Participants

Twelve healthy volunteers with normal or corrected-to-normal vision were recruited from the Beijing Normal University. Eligible participants indicated via a questionnaire that they did not have panic disorder. Participants majoring in film studies were also excluded.

1.2. Neutral faces rating procedure

Before the experiment, participants completed two practice trials to familiarize themselves with the procedure, using films different from those in the formal experiment. Participants initiated the session in each trial by viewing a 2-second clip of a neutral face rendered in grayscale. After the presentation of the clips, participants were tasked with evaluating the emotions portrayed by the actor in neutral faces using a scale. The scale involved rating the valence of the neutral face on a scale from -4 to 4, where -4 denoted a negative emotion, and 4 indicated a positive emotion. The rating had a 3.5-second response time. Subsequently, a 2-second inter-trial interval (ITI) concluded the trial, and participants seamlessly progressed to the subsequent trial, completing 30 trials. Stimuli delivery and response recording were controlled using PsychoPy 3.2 software (https://www.psychopy.org/), administered on a 14-inch laptop.

1.3. Emotional scenes rating procedure

Before the experiment, participants completed two practice trials to familiarize themselves with the procedure, using films different from those in the formal experiment. Participants initiated the session in each trial by viewing a 4-second clip of an emotional scene rendered in grayscale. Following the clip presentation, participants were asked to judge the feeling of the emotional scene with a scale. The scale involved rating the valence of the emotional scene on a scale from -4 to 4, where -4 denoted a negative feeling, and 4 indicated a positive feeling. The rating had a 3.5-second response time. Subsequently, a 2-second ITI concluded the trial, and participants seamlessly progressed to the subsequent trial, completing 30 trials. Stimuli delivery and response recording were controlled using PsychoPy 3.2 software, administered on a 14-inch laptop.

1.4. Data Analysis

For the neutral faces rating experiment, our hypothesis posits that all neutral faces exhibit similar valence, and the averaged valence of faces approaches zero. To test this, we calculated the average facial valence under each type of emotional scene. Subsequently, a repeated-measure analysis of variance (ANOVA) was conducted to confirm no statistical differences in valence among the three emotional conditions.

In the emotional scenes rating experiment, our hypothesis posits that fearful scenes have a negative valence, neutral scenes have a neutral valence and happy scenes have a positive valence. To test this, we computed the average valence for all scenes under each type of emotional scene. Subsequently, a repeated-measure ANOVA was conducted to confirm the statistical differences in valence for scenes among the three emotional conditions.
