# Supplementary material for: Reexamining the Kuleshov effect: Behavioral and neural evidence from authentic film experiments
Source: PLoS One. 2024 Aug 5;19(8):e0308295. doi: 10.1371/journal.pone.0308295 (PMC11299807; doi:10.1371/journal.pone.0308295)
Supplement: S2 Text — Comparison of brain activity for Face_2 between emotional and neutral conditions. (DOCX) [file pone.0308295.s002.docx]

**Reexamining the Kuleshov effect: behavioral and neural evidence from authentic film experiments**

*fMRI Contrasting Results*

**Supplementary results**

***Direct comparison of Face_2 between fearful or happy condition and neutral condition***

Examining the bars corresponding to the fearful and happy conditions in Fig 3C, it becomes evident that the Kuleshov effect introduces a noticeable context-dependent bias in emotional perception. This effect alters the interpretation of neutral faces based on accompanying emotional scenes. Our subsequent analysis aimed to probe the neural correlates of this Kuleshov effect bias, focusing on how neutral faces exhibit distinct brain activation patterns when preceded by fearful or happy scenes [1].

In the fearful condition compared to the neutral condition, the contrast between Face_2 revealed significant activation in 17 clusters, involving regions such as the bilateral cerebellum, fusiform gyrus (FG), parahippocampal gyrus (PHC), angular gyrus (AG), PCC, cuneus, precuneus, precentral gyrus, and postcentral gyrus (for additional AAL atlas labels, S4 Table and S7A Fig). These regions are well-established in facial and emotional processing, supporting that the Kuleshov effect reflects a contextual modulation of face perception.

Conversely, when contrasting Face_2 in the happy condition with those in the neutral condition, only one cluster of significant activation was detected, encompassing the right cuneus, right precuneus, and right calcarine fissure and surrounding cortex (CAL) (S4 Table and S7B Fig). These regions are linked to visual processing, suggesting that the influence of happy scenes on neutral faces is comparatively weaker than fearful scenes.

# **References**

1. Mobbs D, Weiskopf N, Lau HC, Featherstone E, Dolan RJ, Frith CD. The Kuleshov Effect: the influence of contextual framing on emotional attributions. Soc Cogn Affect Neurosci. 2006;1: 95–106. doi:10.1093/scan/nsl014
