# Supplementary figures and images for: Reexamining the Kuleshov effect: Behavioral and neural evidence from authentic film experiments
Source: PLoS One. 2024 Aug 5;19(8):e0308295. doi: 10.1371/journal.pone.0308295 (PMC11299807; doi:10.1371/journal.pone.0308295)

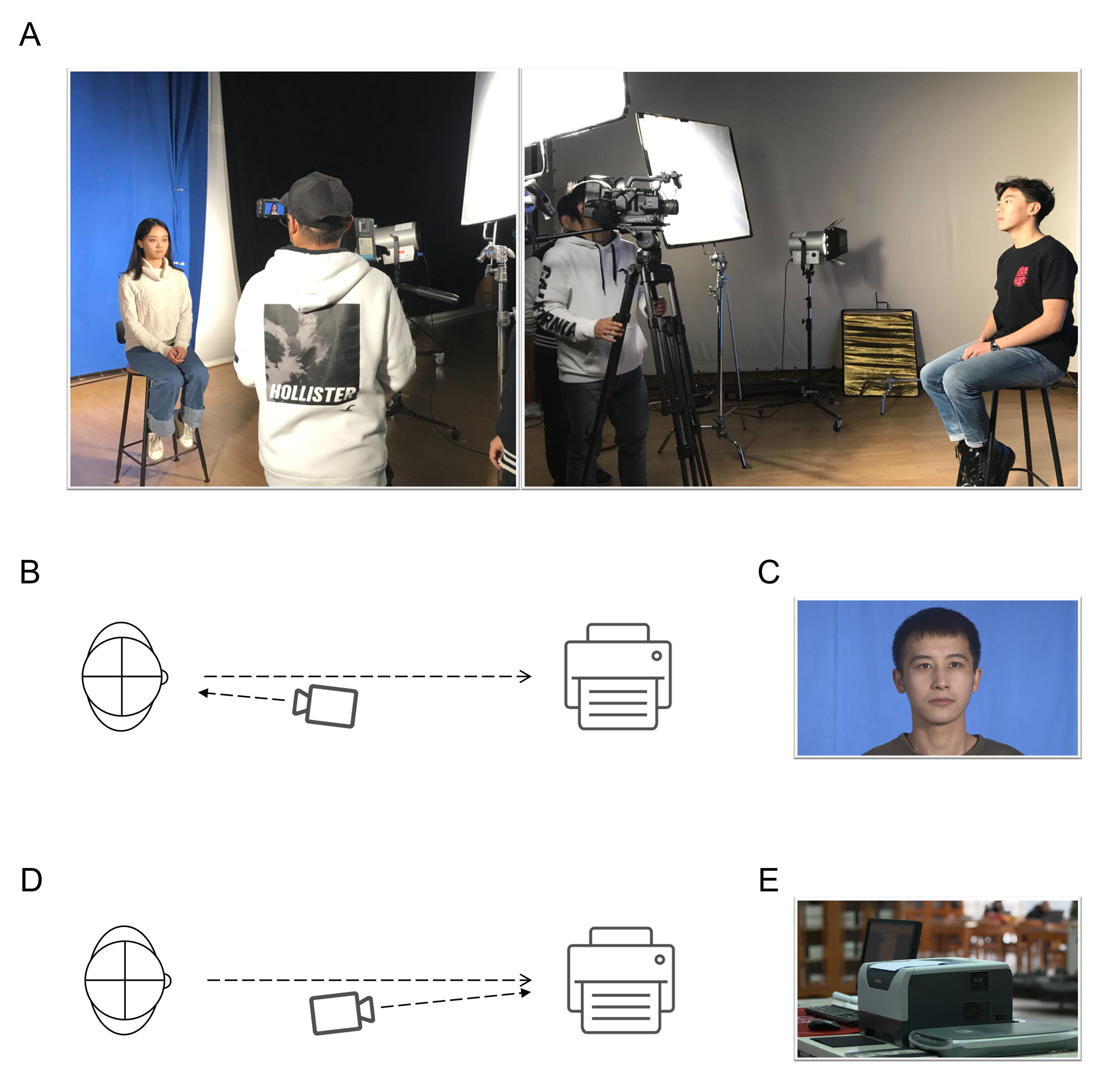

Supplement: S1 Fig — This figure illustrates the process of shooting film clips. We employed a cinecamera, lighting equipment, and a blue screen for shooting the film clips (A). Filming encompassed the sequential capture of a neutral face and an emotional scene within a face-scene-face sequence employing the shot-reverse-shot structure. The shot of the neutral face was taken with the camera positioned close to the 180-degree axis, facing the face (B). The actor was directed to look at a cross marker before them (C). As for the reverse shot of the emotional scene, the camera was placed near the 180-degree axis, oriented toward the object (D) and (E). (TIF) [file pone.0308295.s003.tif]

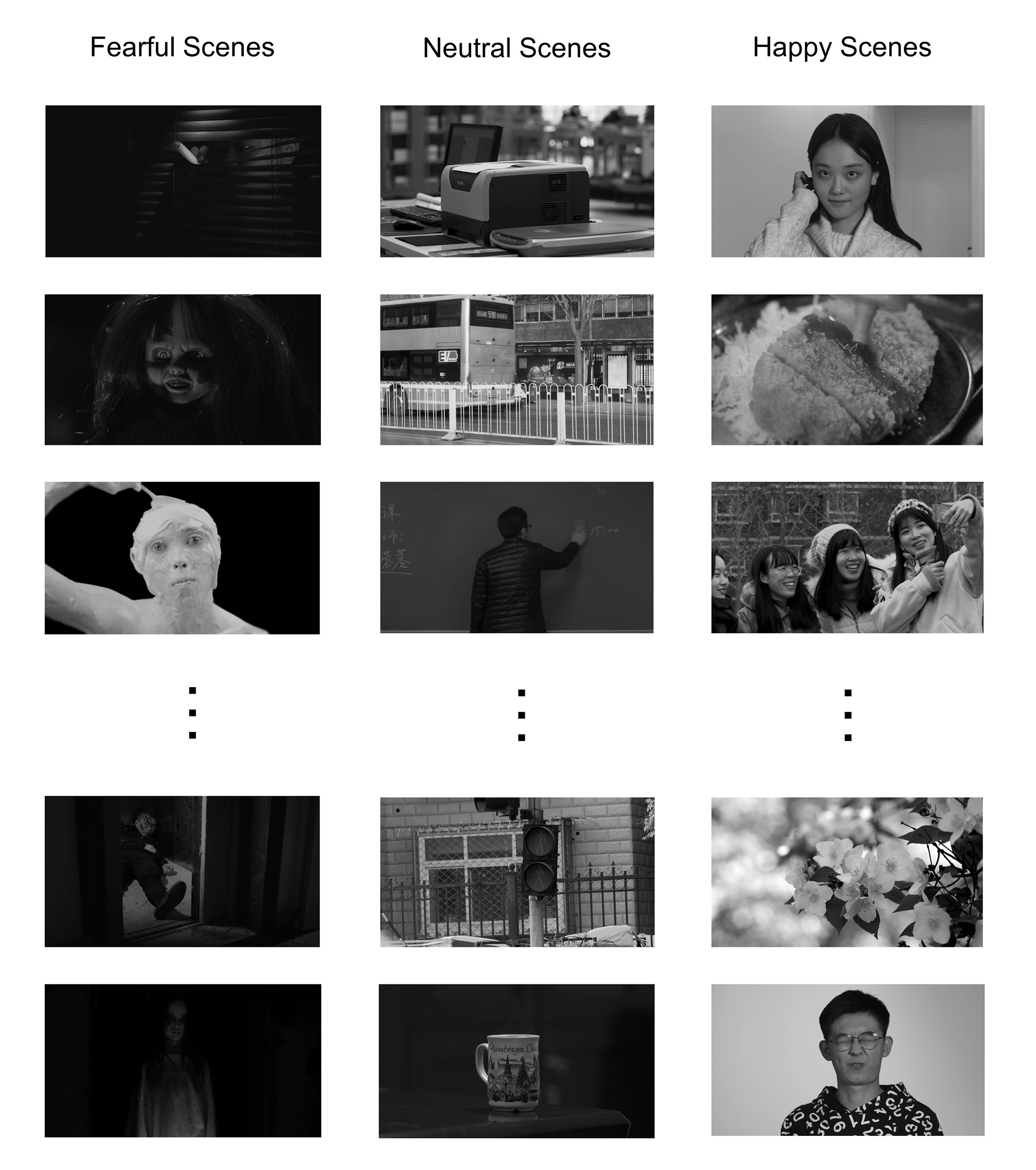

Supplement: S2 Fig — These scenarios spanned various genres, including horror, documentary, and comedy. Fearful scenes depicted chilling scenarios such as peeping, murder, and a ghost. Neutral scenes featured commonplace objects like a printer, a bus stop, and a cup. Happy scenes showcased joyful images of an attractive girl, food, and humorous expressions. (TIF) [file pone.0308295.s004.tif]

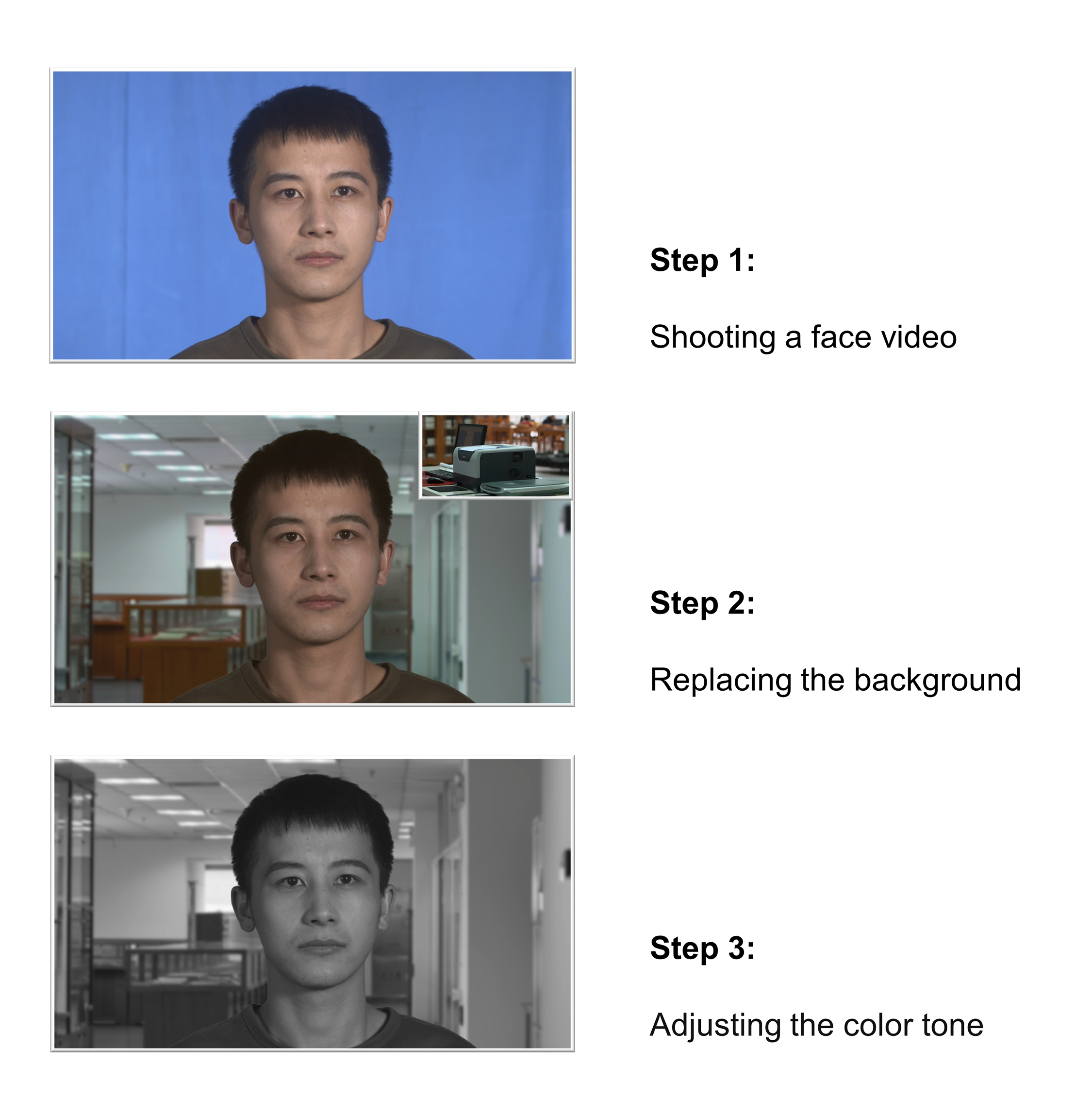

Supplement: S3 Fig — The current figure outlines the steps involved in creating the film clips. The process consists of three main steps for generating the neutral face material. First, we recorded a video featuring a facial shot without expression. Second, the background was substituted with a surrounding video or an image, all of which was shot along with an emotional scene. And, adjustments were made to the color temperature, brightness, and contrast of the facial videos to harmonize them with the emotional scenes. Lastly, we converted the color facial video into black and white [19]. Regarding the reverse shot, namely the emotional scene, we simply applied a black-and-white filter to the footage. (TIF) [file pone.0308295.s005.tif]

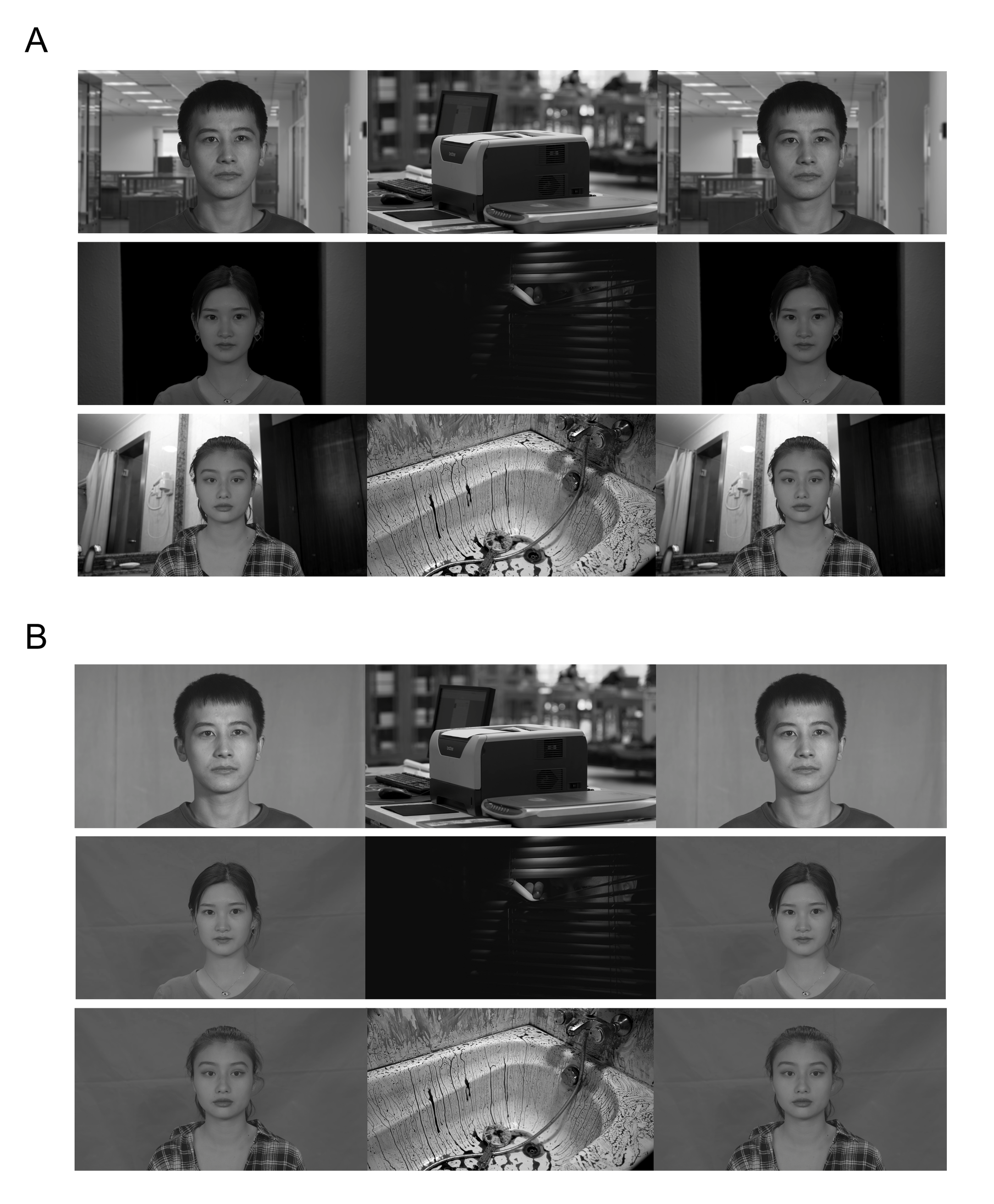

Supplement: S4 Fig — This figure compares highly authentic film clips and those with low ecological validity. Previous studies utilized static images for neutral faces [19] or used unmatched backgrounds with reverse shots [18]. These approaches significantly differ from authentic films. In contrast to the lower authentic film clips displayed in (A), neutral faces with replaced backgrounds (B) provide a more authentic and immersive experience. Therefore, there is a compelling need to reevaluate the Kuleshov effect using highly authentic films. (TIF) [file pone.0308295.s006.tif]

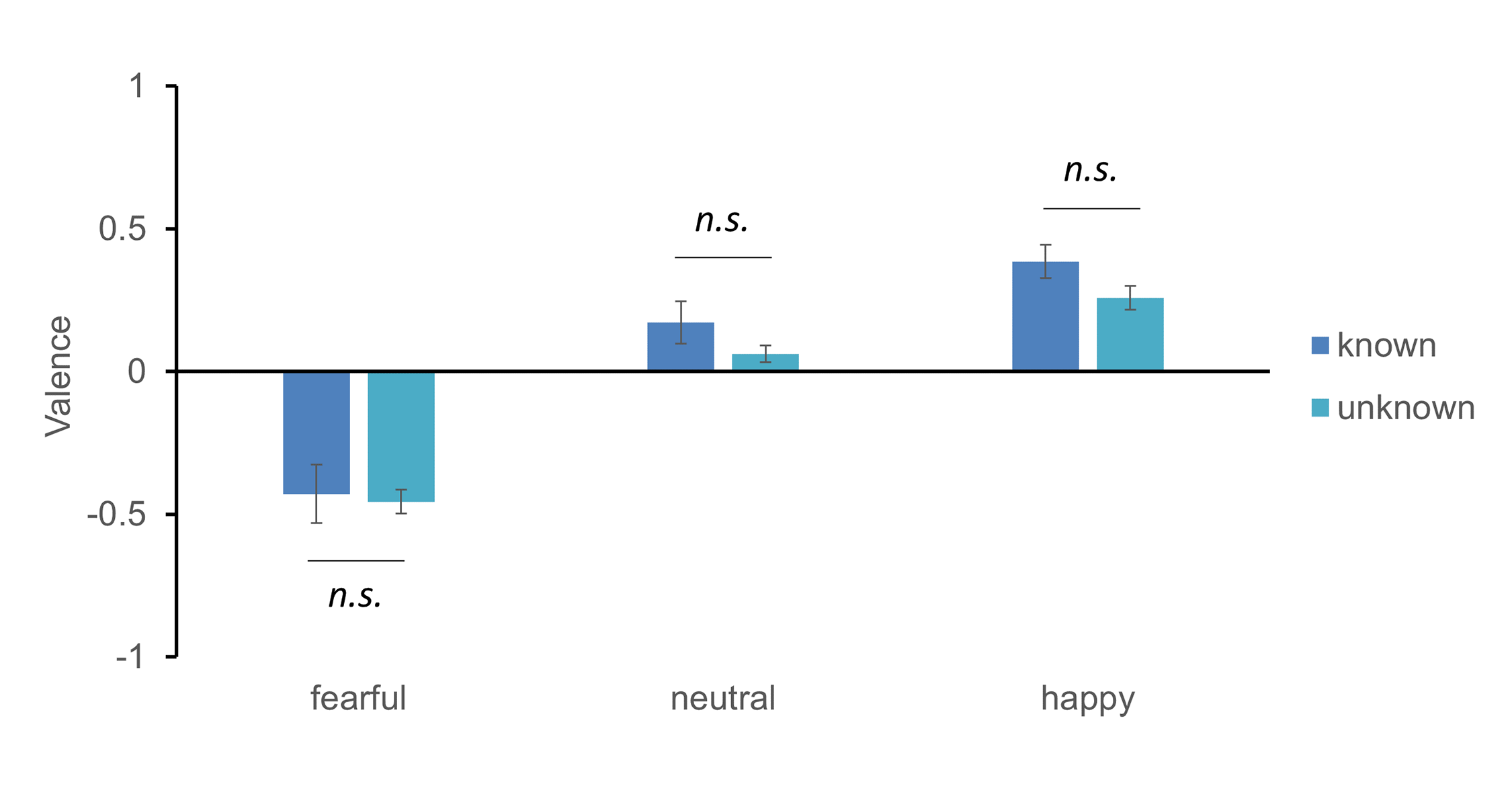

Supplement: S5 Fig — To identify and exclude participants with prior knowledge of the Kuleshov effect, we administered a knowledge test. Out of the 59 participants, seven already knew about the Kuleshov effect, while 52 did not. An Independent Samples Mann-Whitney U Test conducted on valence ratings across these two groups (unknown, known) revealed no significant differences between the groups, either in the fearful condition, neutral condition, or happy condition (all p > 0.1). This suggests that prior knowledge of the Kuleshov effect does not affect the observation of the Kuleshov effect. (TIF) [file pone.0308295.s007.tif]

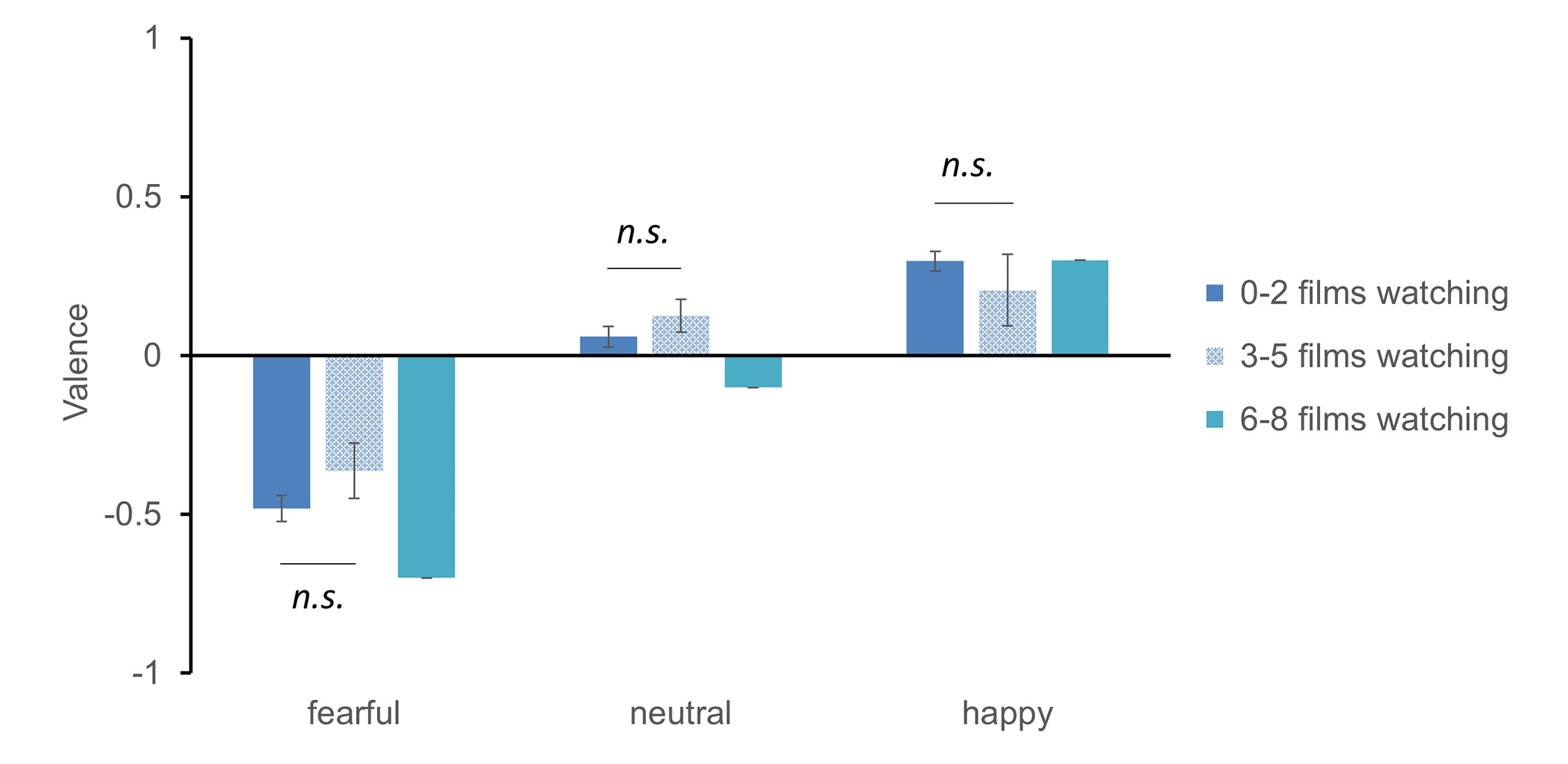

Supplement: S6 Fig — To examine whether film-watching frequency influences the observation of the Kuleshov effect, we conducted a subgroup comparison based on watching frequency. Of the 59 participants, 42 watched 0–2 films per week, 16 watched 3–5 films per week, and only one participant watched 6–8 films per week. Although there is a tendency for the middle-watching group (3–5 films per week) to have lower valence than the less-watching group (0–2 films per week), an Independent Samples Mann-Whitney U Test conducted on valence ratings across the two groups (0–2 films per week, 3–5 films per week) revealed no significant differences between the groups, either in the fearful condition, neutral condition, or happy condition (all p > 0.1). This suggests that film-watching frequency does not significantly affect the perception of the Kuleshov effect. (TIF) [file pone.0308295.s008.tif]

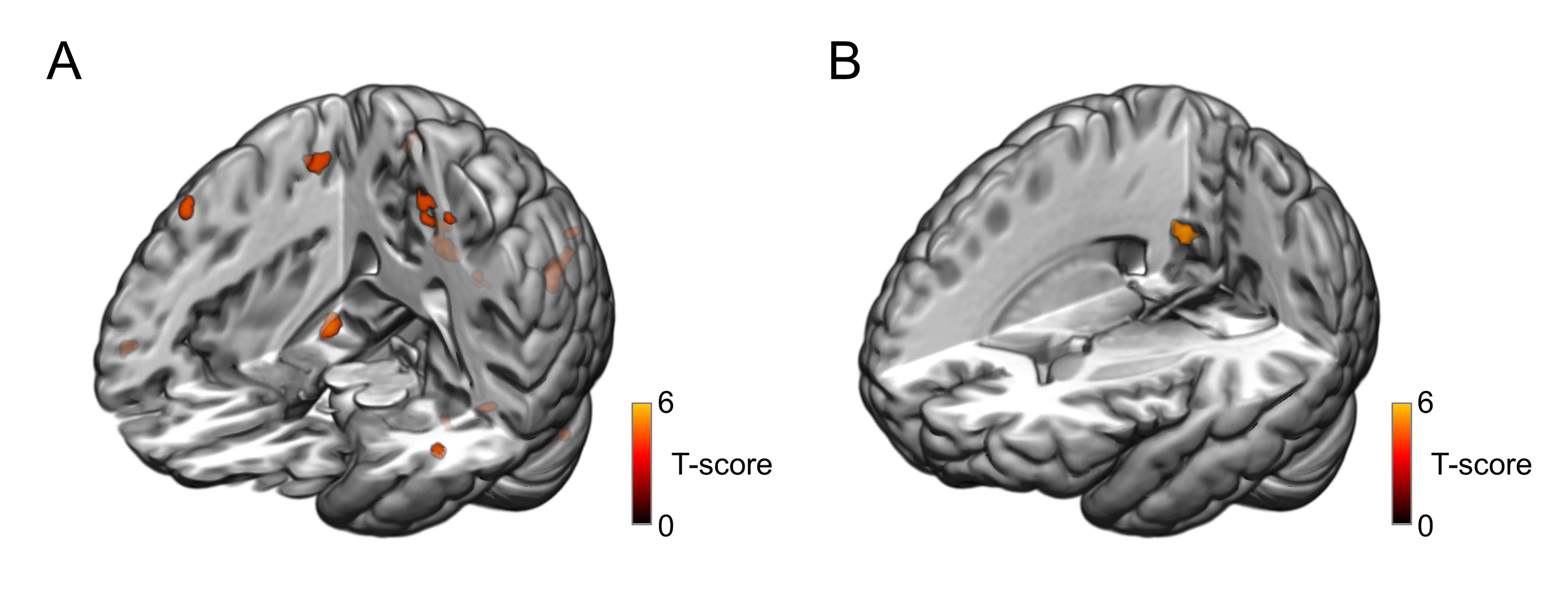

Supplement: S7 Fig — (A) Brain activity was obtained by subtracting Face_2 in the neutral condition from Face_2 in the fearful condition. (B) Brain activity was obtained by subtracting Face_2 in the neutral condition from Face_2 in the happy condition. (p < 0.05, FDR-corrected, cluster size > 5 voxels). (TIF) [file pone.0308295.s009.tif]
