# Supplementary material for: Reexamining the Kuleshov effect: Behavioral and neural evidence from authentic film experiments
Source: PLoS One. 2024 Aug 5;19(8):e0308295. doi: 10.1371/journal.pone.0308295 (PMC11299807; doi:10.1371/journal.pone.0308295)
Supplement: S1 Table — To uncover the neural correlates associated with the new meaning attributed to the second face, our fMRI analysis compared brain activity between Face_2 and Face_1 in fearful condition. (p < 0.05, FDR-corrected, cluster size > 5 voxels). (DOCX) [file pone.0308295.s010.docx]

**S1 Table. fMRI Results: Face_2 minus Face_1 in fearful condition.**

| **Brain Region** | **AAL Atlas Labels** | **Peak Voxel Coordinate (MNI)** | **Cluster Size (KE)** | **T-score** |
| --- | --- | --- | --- | --- |
| ***Face_2 > Face_1*** *(FDR-corrected cluster threshold, p < 0.05)* | | | | |
| Cerebellum | Cerebellum_9_L Cerebellum_9_R | 0, -46, -52 | 97 | 6.579 |
| Cerebellum | Cerebellum_8_R Cerebellum_9_R Cerebellum_7b_R | 26, -48, -52 | 396 | 6.737 |
| Cerebellum | Cerebellum_8_L Cerebellum_9_L | -14, -62, -52 | 191 | 5.961 |
| Cerebellum | Cerebellum_6_L Cerebellum_Crus2_L Cerebellum_4_5_L Vermis_4_5 Cerebellum_4_5_R Cerebellum_6_R Vermis_6 Cerebellum_Crus1_R Cerebellum_Crus1_L Cerebellum_Crus2_R Cerebellum_7b_R Cerebellum_7b_L Vermis_3 Cerebellum_8_R Vermis_7 | -44, -72, -42 | 2428 | 7.769 |
| Cerebellum | Cerebellum_Crus2_R Cerebellum_Crus1_R | 36, -62, -42 | 12 | 3.204 |
| Cerebellum | Vermis_10 | 4, -46, -28 | 19 | 3.374 |
| Cerebellum | Cerebellum_6_R | 28, -40, -34 | 5 | 2.965 |
| Cerebellum | Cerebellum_8_R Vermis_8 Cerebellum_Crus2_R | 6, -66, -32 | 7 | 3.174 |
| Cerebellum | Cerebellum_Crus1_L | -32, -66, -32 | 30 | 4.594 |
| Right Inferior Temporal Gyrus | Temporal_Inf_R | 54, -16, -34 | 9 | 3.876 |
| Left Inferior Temporal Gyrus | Temporal_Inf_L | -52, -16, -34 | 5 | 3.599 |
| SMA/Angular Gyrus/Insula/STG/ACC  /Hippocampus (bilaterally) | Postcentral_R Postcentral_L Precentral_R Parietal_Inf_L Parietal_Inf_R Precentral_L Angular_R Frontal_Sup_2_R Paracentral_Lobule_L Supp_Motor_Area_R Supp_Motor_Area_L Parietal_Sup_L Precuneus_R Parietal_Sup_R SupraMarginal_R Rolandic_Oper_R Rolandic_Oper_L Cingulate_Mid_R Paracentral_Lobule_R Temporal_Inf_R Angular_L Temporal_Mid_R Precuneus_L Frontal_Sup_2_L Frontal_Mid_2_R Frontal_Inf_Oper_R Insula_R Temporal_Sup_L Cingulate_Mid_L SupraMarginal_L Temporal_Sup_R Insula_L Heschl_R Heschl_L Cuneus_R Occipital_Mid_R Caudate_L Cingulate_Post_L Thal_PuM_R Frontal_Inf_Oper_L Caudate_R Occipital_Sup_R Hippocampus_L Occipital_Mid_L Temporal_Pole_Sup_L Cingulate_Post_R Hippocampus_R Temporal_Pole_Sup_R Thal_PuM_L Putamen_R Thal_PuA_R Occipital_Sup_L | 18, -38, 18 | 20624 | 9.815 |
| Cerebellum | Vermis_7 Cerebellum_Crus2_L | 0, -80, -26 | 20 | 3.785 |
| Left Temporal Lobe | Temporal_Mid_L Temporal_Inf_L | -66, -26, -14 | 55 | 3.527 |
| Right Frontal Lobe | OFCant_R Frontal_Mid_2_R OFCmed_R | 28, 46, -14 | 111 | 5.299 |
| Right Temporal Lobe | Temporal_Inf_R | 60, -52, -16 | 7 | 3.581 |
|  | OFCant_L OFClat_L Frontal_Mid_2_L | -26, 46, -14 | 26 | 4.119 |
| Right Temporal Lobe | Temporal_Mid_L | -54, -32, -8 | 12 | 3.323 |
| Left Frontal Lobe | Frontal_Sup_2_L | -26, 58, -10 | 7 | 3.201 |
| Right Temporal Lobe | Temporal_Sup_R Insula_R Temporal_Pole_Sup_R | 48, -4, -4 | 50 | 4.475 |
| Right Frontal Lobe | Frontal_Mid_2_R Frontal_Sup_2_R Frontal_Inf_Tri_R | 42, 46, 22 | 738 | 6.363 |
| Left Temporal Lobe | Temporal_Sup_L | -40, -26, 2 | 13 | 3.846 |
| Right Frontal Lobe | Frontal_Sup_2_R | 26, 66, -2 | 19 | 3.755 |
| Right Temporal Lobe | Temporal_Sup_R | 60, -16, 6 | 8 | 3.734 |
| Right SFG | Frontal_Sup_2_R | 32, 64, 10 | 12 | 3.410 |
| Left STG | Temporal_Sup_L | -44, -34, 8 | 47 | 4.748 |
| Left IFG | Frontal_Inf_Tri_L Frontal_Mid_2_L | -36, 42, 12 | 7 | 3.173 |
| Caudate | Caudate_R | 20, 24, 14 | 12 | 3.767 |
| Left Middle Frontal Sulcus | Frontal_Mid_2_L | -40, 48, 20 | 5 | 2.835 |
| Cuneus | Cuneus_L | 2, -88, 34 | 13 | 4.016 |
| Left Precentral Gyrus | Precentral_L | -60, 2, 26 | 25 | 3.572 |
| Right DMPFC | Frontal_Mid_2_R Frontal_Sup_2_R | 26, 56, 32 | 45 | 3.652 |
| Right ACC | Cingulate_Mid_R Frontal_Sup_Medial_L ACC_sup_R | 2, 36, 32 | 32 | 3.509 |
| Left Middle Frontal Sulcus | Frontal_Mid_2_L | -38, 44, 30 | 6 | 2.856 |
| Precuneus | Cuneus_L Precuneus_L | -8, -76, 32 | 19 | 3.492 |
| Precuneus | Precuneus_L | -10, -66, 40 | 33 | 4.189 |
| Left VMPFC | Frontal_Sup_Medial_L | 0, 28, 40 | 15 | 3.620 |
| Left MFG | Frontal_Mid_2_L | -32, 22, 50 | 5 | 2.976 |
