# Supplementary material for: Reexamining the Kuleshov effect: Behavioral and neural evidence from authentic film experiments
Source: PLoS One. 2024 Aug 5;19(8):e0308295. doi: 10.1371/journal.pone.0308295 (PMC11299807; doi:10.1371/journal.pone.0308295)
Supplement: S2 Table — To uncover the neural correlates associated with the new meaning attributed to the second face, our fMRI analysis compared brain activity between Face_2 and Face_1 in happy condition. (p < 0.05, FDR-corrected, cluster size > 5 voxels). (DOCX) [file pone.0308295.s011.docx]

**S2 Table. fMRI Results: Face_2 minus Face_1 in happy condition.**

| **Brain Region** | **AAL Atlas Labels** | **Peak Voxel Coordinate (MNI)** | **Cluster Size (KE)** | **T-score** |
| --- | --- | --- | --- | --- |
| ***Face_2 > Face_1*** *(FDR-corrected cluster threshold, p < 0.05)* | | | | |
| Cerebellum | Cerebellum_9_L | -10, -40, -56 | 19 | 5.364 |
| Cerebellum | Cerebellum_6_L Cerebellum_4_5_L Cerebellum_6_R Cerebellum_Crus1_L Cerebellum_Crus2_L Vermis_4_5 Cerebellum_8_L Cerebellum_4_5_R Vermis_6 Cerebellum_8_R Cerebellum_Crus1_R Cerebellum_7b_L Cerebellum_9_L Cerebellum_Crus2_R Cerebellum_7b_R Vermis_7 Cerebellum_9_R Lingual_L Vermis_8 Cerebellum_3_R Vermis_3 Lingual_R Cerebellum_10_L | -26, -50, -26 | 3891 | 8.026 |
| Cerebellum | Cerebellum_8_R Cerebellum_Crus2_R Cerebellum_7b_R Vermis_8 Cerebellum_9_R | 14, -62, -44 | 219 | 4.855 |
| Cerebellum | Vermis_8 Cerebellum_8_L Vermis_7 Cerebellum_Crus2_L | -2, -68, -34 | 52 | 5.447 |
| Right Temporal Lobe | Temporal_Inf_R Temporal_Mid_R | 64, -24, -24 | 525 | 8.769 |
| Left Temporal Lobe | Temporal_Inf_L Temporal_Mid_L | -56, -46, -14 | 57 | 4.084 |
| Cerebellum | Vermis_3 Cerebellum_3_L | 4, -40, -18 | 11 | 3.199 |
| SMA/Angular Gyrus/STG/ACC  /Hippocampus (bilaterally) | Frontal_Mid_2_R Frontal_Sup_2_R Parietal_Inf_R Parietal_Inf_L Postcentral_R Precuneus_R Angular_R SupraMarginal_R Parietal_Sup_R Supp_Motor_Area_R Precentral_R Cingulate_Mid_R Parietal_Sup_L Postcentral_L ACC_sup_R ACC_pre_R Supp_Motor_Area_L Paracentral_Lobule_L ACC_sup_L Precuneus_L Cuneus_R Frontal_Sup_Medial_L Angular_L Cingulate_Mid_L ACC_pre_L Frontal_Sup_Medial_R Caudate_R Caudate_L Occipital_Mid_R Precentral_L SupraMarginal_L OFCant_R Frontal_Sup_2_L Occipital_Mid_L Frontal_Med_Orb_R Occipital_Sup_R Paracentral_Lobule_R Frontal_Inf_Tri_R Frontal_Inf_Orb_2_R OFCmed_R Hippocampus_L Calcarine_L Thal_PuM_L Thal_PuM_R ACC_sub_R Cuneus_L | 18, -38, 16 | 14467 | 8.754 |
| Left Frontal Lobe | Frontal_Sup_2_L Frontal_Mid_2_L OFCant_L | -28, 50, -10 | 116 | 4.676 |
| Right Insula | Insula_R Frontal_Inf_Orb_2_R | 34, 20, -8 | 12 | 3.410 |
| Left Rolandic operculum/Insula/Heschl | Rolandic_Oper_L Insula_L SupraMarginal_L Frontal_Inf_Oper_L Temporal_Pole_Sup_L Postcentral_L Temporal_Sup_L Precentral_L Heschl_L | -44, 0, 12 | 503 | 5.912 |
| Right Rolandic operculum/Insula/Heschl | Rolandic_Oper_R Insula_R Heschl_R SupraMarginal_R Temporal_Sup_R Postcentral_R | 50, -20, 20 | 274 | 5.619 |
| Right Rolandic operculum/Insula/Heschl | Frontal_Inf_Oper_R Rolandic_Oper_R Insula_R Precentral_R Temporal_Sup_R Temporal_Pole_Sup_R Frontal_Inf_Tri_R Putamen_R Heschl_R | 40, 4, 2 | 840 | 6.420 |
| Precuneus | Precuneus_R Calcarine_R | 32, -48, 0 | 89 | 7.648 |
| Left STG | Temporal_Sup_L | -42, -24, 0 | 6 | 3.217 |
| Putamen | Putamen_R | 30, -2, 2 | 7 | 3.651 |
| Left MFG/Insula | Frontal_Mid_2_L Frontal_Inf_Tri_L Insula_L | -20, 34, 10 | 85 | 4.571 |
| Left MFG | Frontal_Mid_2_L Frontal_Inf_Tri_L | -40, 44, 16 | 53 | 3.434 |
| Calcarine | Calcarine_L | -16, -62, 12 | 5 | 2.969 |
| Putamen | Putamen_R | 28, -12, 10 | 7 | 3.517 |
| Insula | Insula_L | -28, 16, 12 | 8 | 3.156 |
| Left Thalamus | Thal_VL_L | -14, -6, 14 | 6 | 3.603 |
| Left STG | Temporal_Sup_L | -44, -40, 14 | 5 | 4.137 |
| Right Heschl | Heschl_R Temporal_Sup_R | 34, -32, 14 | 17 | 3.820 |
| Right STG | Temporal_Sup_R Rolandic_Oper_R | 46, -32, 18 | 5 | 3.234 |
| Right Thalamus | Thal_PuM_R | 6, -20, 16 | 7 | 3.232 |
| Precuneus/Cuneus | Precuneus_L Cuneus_L Calcarine_L Occipital_Sup_L Parietal_Sup_L | -12, -66, 28 | 332 | 4.594 |
| Left Supramarginal Gyrus | SupraMarginal_L | -68, -26, 20 | 7 | 3.291 |
| ACC (bilaterally) | Cingulate_Mid_R Cingulate_Mid_L Precuneus_R Cingulate_Post_R Cingulate_Post_L Paracentral_Lobule_R Precuneus_L Paracentral_Lobule_L | 8, -30, 42 | 661 | 6.6123 |
| Left Precentral Gyrus | Frontal_Inf_Oper_L Precentral_L | -38, -2, 24 | 13 | 4.638 |
| Cuneus | Cuneus_L | 0, -88, 34 | 8 | 3.592 |
| Left ACC | Cingulate_Mid_L ACC_sup_L | -8, 10, 34 | 40 | 3.612 |
| Right MFG | Frontal_Mid_2_R | 30, 26, 32 | 8 | 3.353 |
| Left MFG | Frontal_Mid_2_L | -36, 34, 42 | 5 | 3.066 |
| Right Precentral Gyrus | Precentral_R Postcentral_R | 38, -20, 46 | 5 | 2.863 |
| Right SMA/ACC | Supp_Motor_Area_R Cingulate_Mid_R | 8, -14, 52 | 9 | 4.154 |
| Left SMA | Supp_Motor_Area_L | -14, -2, 50 | 10 | 3.322 |
| Precuneus | Precuneus_L Precuneus_R | -2, -72, 60 | 15 | 3.742 |
| Precuneus | Precuneus_L Parietal_Sup_L | -12, -68, 64 | 5 | 2.837 |
| Precuneus | Precuneus_R Paracentral_Lobule_R | 4, -46, 64 | 5 | 2.860 |
| Left Precentral Gyrus | Precentral_L Postcentral_L | -22, -24, 76 | 17 | 3.377 |
