# Supplementary material for: Reexamining the Kuleshov effect: Behavioral and neural evidence from authentic film experiments
Source: PLoS One. 2024 Aug 5;19(8):e0308295. doi: 10.1371/journal.pone.0308295 (PMC11299807; doi:10.1371/journal.pone.0308295)
Supplement: S3 Table — To uncover the neural correlates associated with the new meaning attributed to the second face, our fMRI analysis compared brain activity between Face_2 and Face_1 in neutral condition. (p < 0.05, FDR-corrected, cluster size > 5 voxels). (DOCX) [file pone.0308295.s012.docx]

**S3 Table. fMRI Results: Face_2 minus Face_1 in neutral condition.**

| **Brain Region** | **AAL Atlas Labels** | **Peak Voxel Coordinate (MNI)** | **Cluster Size (KE)** | **T-score** |
| --- | --- | --- | --- | --- |
| ***Face_2 > Face_1*** *(FDR-corrected cluster threshold, p < 0.05)* | | | | |
| Cerebellum | Cerebellum_8_R Cerebellum_7b_R Cerebellum_9_R Vermis_8 Cerebellum_Crus2_R | 30, -50, -54 | 483 | 7.099 |
| Cerebellum | Cerebellum_6_L Cerebellum_6_R Cerebellum_Crus1_L Cerebellum_4_5_L Cerebellum_8_L Vermis_4_5 Vermis_6 Cerebellum_Crus2_L Cerebellum_4_5_R Cerebellum_Crus1_R Cerebellum_7b_L Vermis_7 Cerebellum_9_L Vermis_3 Vermis_8 Cerebellum_3_R | -4, -62, -18 | 3521 | 9.957 |
| Cerebellum | Cerebellum_9_L | -4, -42, -52 | 5 | 3.670 |
| Cerebellum | Cerebellum_Crus1_L Cerebellum_Crus2_L | -46, -70, -36 | 9 | 4.263 |
| Cerebellum | Vermis_8 Cerebellum_8_R Vermis_7 | 6, -66, -36 | 16 | 3.179 |
| Cerebellum | Cerebellum_8_L Vermis_8 | -4, -66, -34 | 11 | 4.541 |
| Right Temporal Lobe | Temporal_Inf_R Temporal_Mid_R | 62, -24, -22 | 329 | 7.421 |
| Left Temporal Lobe | Temporal_Mid_L Temporal_Inf_L | -62, -34, -16 | 14 | 3.514 |
| Left Temporal Lobe | Temporal_Inf_L | -52, -28, -18 | 6 | 2.901 |
| Right Insula/Putamen/Heschl  /STG | Insula_R Frontal_Inf_Oper_R Rolandic_Oper_R Putamen_R Precentral_R Temporal_Sup_R Temporal_Pole_Sup_R Heschl_R Frontal_Inf_Tri_R Pallidum_R | 36, 2, 8 | 1928 | 9.426 |
| Right OFC | Frontal_Mid_2_R OFCant_R Frontal_Sup_2_R OFCmed_R | 34, 54, -12 | 151 | 5.005 |
| Left Insula/STG/Heschl  /Hippocampus/Angular Gyrus/Thalamus | Postcentral_L Parietal_Inf_L SupraMarginal_L Insula_L Rolandic_Oper_L Parietal_Sup_L Temporal_Sup_L Frontal_Inf_Oper_L Precuneus_L Precentral_L Temporal_Pole_Sup_L Caudate_L Heschl_L Putamen_L Precuneus_R Thal_VPL_L Cingulate_Post_L Hippocampus_L Thal_PuM_L Angular_L Frontal_Inf_Orb_2_L | -30, -56, 2 | 5972 | 7.862 |
| Right Insula | Insula_R Frontal_Inf_Orb_2_R OFCpost_R | 36, 20, -10 | 49 | 4.816 |
| Left SFG/OFC | Frontal_Sup_2_L Frontal_Mid_2_L OFCant_L | -22, 46, -2 | 93 | 4.418 |
| Left Insula/STG | Temporal_Sup_L Insula_L | -38, -14, -6 | 16 | 3.428 |
| Right ACC/SFG/Hippocampus  /Precuneus/STG  /Precentral Gyrus | Postcentral_R SupraMarginal_R Parietal_Inf_R Frontal_Mid_2_R Frontal_Sup_2_R Parietal_Sup_R Cingulate_Mid_R Supp_Motor_Area_R ACC_sup_R ACC_sup_L Supp_Motor_Area_L Cingulate_Mid_L Angular_R Precentral_R Rolandic_Oper_R ACC_pre_R ACC_pre_L Precuneus_R Paracentral_Lobule_L Temporal_Sup_R Frontal_Sup_Medial_L Frontal_Sup_2_L Precentral_L Frontal_Sup_Medial_R Caudate_R Cuneus_R Heschl_R Cingulate_Post_L Frontal_Med_Orb_R Hippocampus_R Cingulate_Post_R ACC_sub_L Occipital_Sup_R Thal_PuM_R Occipital_Mid_R | 44, -36, 54 | 11662 | 8.370 |
| Left Frontal Lobe | Frontal_Sup_2_L Frontal_Mid_2_L | -32, 46, 0 | 6 | 3.408 |
| Left Heschl/Insual | Heschl_L Insula_L Temporal_Sup_L | -38, -24, 4 | 48 | 3.689 |
| Caudate | Caudate_R | 16, 24, 2 | 7 | 4.087 |
| Right SFG | Frontal_Sup_2_R | 24, 58, 4 | 25 | 3.479 |
| Putamen | Putamen_L | -26, -6, 2 | 9 | 3.511 |
| Right Heschl | Heschl_R Temporal_Sup_R | 38, -26, 8 | 21 | 4.337 |
| Left Middle Frontal Sulcus | Frontal_Mid_2_L Frontal_Inf_Tri_L | -36, 38, 22 | 230 | 5.499 |
| Right Heschl | Heschl_R | 34, -28, 10 | 7 | 3.049 |
| Caudate | Caudate_R | 24, 12, 16 | 41 | 5.263 |
| Caudate | Caudate_L | -18, -8, 18 | 5 | 2.957 |
| Left SFG | Frontal_Sup_2_L | -18, 48, 20 | 6 | 2.992 |
| Cuneus | Cuneus_L Precuneus_L | -4, -74, 30 | 68 | 3.875 |
| Caudate | Caudate_R | 24, -4, 32 | 59 | 4.379 |
| Left Precentral Gyrus | Precentral_L Frontal_Inf_Oper_L | -54, 4, 30 | 27 | 3.630 |
| Left SFG | Frontal_Sup_2_L | -26, 26, 30 | 7 | 3.579 |
| Left Angular Gyrus | Angular_L Parietal_Inf_L Parietal_Sup_L Occipital_Mid_L | -36, -60, 40 | 73 | 3.508 |
| Right MFG | Frontal_Mid_2_R | 38, 32, 38 | 101 | 4.150 |
| Right Posterior Cingulate Cortex/SMA | Cingulate_Mid_R Paracentral_Lobule_R Cingulate_Mid_L Supp_Motor_Area_R Precuneus_R | 10, -28, 42 | 366 | 5.813 |
| Right SFG | Frontal_Sup_2_R | 24, 34, 38 | 5 | 3.159 |
| Right SFG | Frontal_Sup_2_R | 20, 18, 38 | 7 | 4.193 |
| Left Precentral Gyrus | Precentral_L | -32, -10, 46 | 29 | 5.251 |
| Right SFG/SMA | Frontal_Sup_2_R Supp_Motor_Area_R Frontal_Sup_Medial_R | 16, 26, 56 | 251 | 5.264 |
| Right Precuneus | Parietal_Sup_R Precuneus_R | 10, -68, 64 | 54 | 3.319 |
| Left Postcentral Gyrus | Postcentral_L | -22, -30, 56 | 5 | 3.231 |
| Right Paracentral Lobule | Paracentral_Lobule_R | 12, -30, 56 | 7 | 3.805 |
| Right Paracentral Lobule | Paracentral_Lobule_R Precuneus_R | 4, -44, 64 | 6 | 3.182 |
| Left Paracentral Lobule | Paracentral_Lobule_L | 0, -22, 80 | 8 | 3.353 |
