# Supplementary material for: Reexamining the Kuleshov effect: Behavioral and neural evidence from authentic film experiments
Source: PLoS One. 2024 Aug 5;19(8):e0308295. doi: 10.1371/journal.pone.0308295 (PMC11299807; doi:10.1371/journal.pone.0308295)
Supplement: S4 Table — To probe the neural correlates of this Kuleshov effect bias, focus on how neutral faces exhibit distinct brain activation patterns when preceded by fearful or happy scenes. (p < 0.05, FDR-corrected, cluster size > 5 voxels). (DOCX) [file pone.0308295.s013.docx]

**S4 Table. fMRI Results: Face_2 in fearful or happy condition minus Face_2 in neutral condition.**

| **Brain Region** | **AAL Atlas Labels** | **Peak Voxel Coordinate (MNI)** | **Cluster Size (KE)** | **T-score** |
| --- | --- | --- | --- | --- |
| ***Fearful > Neutral*** *(FDR-corrected cluster threshold, p < 0.05)* | | | | |
| Right Cerebellum | Cerebellum_9_L | -2, -52, -40 | 6 | 5.259 |
| Left Cerebellum | Cerebellum_Crus2_L Cerebellum_Crus1_L | -40, -72, -36 | 8 | 4.878 |
| Left Fusiform Gyrus | Fusiform_L | -36, -46, -20 | 7 | 5.232 |
| Left Middle Temporal Gyrus | Temporal_Mid_L | -52, -12, -18 | 9 | 6.367 |
| Right ParaHippocampal Gyrus | ParaHippocampal_R Fusiform_R | 34, -32, -14 | 27 | 7.138 |
| Right Lingual Gyrus | Lingual_R Occipital_Inf_R | 26, -88, -14 | 6 | 4.729 |
| Right VLPFC | Frontal_Inf_Orb_2_R | 52, 40, -6 | 11 | 6.107 |
| Left Precuneus | Precuneus_L Calcarine_L | -10, -60, 16 | 5 | 5.144 |
| Right Precuneus | Precuneus_R Cuneus_R Calcarine_R | 10, -62, 24 | 21 | 5.190 |
| Left Posterior Cingulate Cortex/Precuneus | Precuneus_L Precuneus_R  Cingulate_Post_L | 0, -56, 20 | 22 | 5.598 |
| Left Angular Gyrus | Angular_L Occipital_Mid_L Temporal_Mid_L | -42, -70, 28 | 39 | 5.180 |
| Left Lateral Occipital Cortex | Occipital_Mid_L | -32, -84, 34 | 8 | 5.168 |
| Right Angular Gyrus | Angular_R | 38, -68, 42 | 11 | 4.607 |
| Precentral Gyrus | Precentral_R Postcentral_R | 30, -22, 52 | 28 | 5.461 |
| Right Superior Frontal Sulcus | Frontal_Sup_2_R Frontal_Mid_2_R | 30, 32, 54 | 16 | 5.802 |
| Left Precentral Gyrus | Precentral_L | -30, -24, 64 | 21 | 5.304 |
| Right Postcentral Gyrus | Precentral_L  Postcentral_L | -38, -26, 62 | 5 | 4.969 |
| ***Happy > Neutral*** *(FDR-corrected cluster threshold, p < 0.05)* | | | | |
| Right Precuneus/Cuneus/CAL | Precuneus_R Cuneus_R Calcarine_R | 18, -56, 20 | 31 | 6.314 |
