# Supplementary material for: Reexamining the Kuleshov effect: Behavioral and neural evidence from authentic film experiments
Source: PLoS One. 2024 Aug 5;19(8):e0308295. doi: 10.1371/journal.pone.0308295 (PMC11299807; doi:10.1371/journal.pone.0308295)
Supplement: Attachment — Submitted filename: Response to Reviewers.docx [file pone.0308295.s023.docx]

PONE-D-23-41138

Reexamining the Kuleshov effect: behavioral and neural evidence from authentic film experiments

PLOS ONE

Dear Dr. Wang,

Thank you for submitting your manuscript to PLOS ONE. After careful consideration, we feel that it has merit but does not fully meet PLOS ONE’s publication criteria as it currently stands. Therefore, we invite you to submit a revised version of the manuscript that addresses the points raised during the review process.

Editor comments. First, I have to apologise for the delay with your manuscript. Despite I asked numerous experts to review, I was able to secure only one referee. In general, it seems that although your topic, cinematic cognition, is of considerable general interest, expert researchers within this speciality field generally do not have expertise in brain imaging. As the journal guidelines mandate a minimum of two expert reviews to proceed further, and since we have received only one at present, I have two options: either to reject your manuscript or to offer you an opportunity to revise it based on the available review. I have opted for the latter. I would like to emphasise, at this point, that we cannot proceed further in the process without finding an additional reviewer with expertise in the neuroimaging field. I would therefore invite you to resubmit your work following a thorough revision and to include a response letter addressing each comment made by Reviewer 1. I would suggest that you provide me with six potential reviewers (including email correspondence) whom you know have the expertise to assess your work adequately (and are likely to review). I wish you good luck with your revisions.

Please submit your revised manuscript by Jun 09 2024 11:59PM. If you will need more time than this to complete your revisions, please reply to this message or contact the journal office at [plosone@plos.org](mailto:plosone@plos.org). When you're ready to submit your revision, log on to https://www.editorialmanager.com/pone/ and select the 'Submissions Needing Revision' folder to locate your manuscript file.

Please include the following items when submitting your revised manuscript:

- A rebuttal letter that responds to each point raised by the academic editor and reviewer(s). You should upload this letter as a separate file labeled 'Response to Reviewers'.
- A marked-up copy of your manuscript that highlights changes made to the original version. You should upload this as a separate file labeled 'Revised Manuscript with Track Changes'.
- An unmarked version of your revised paper without tracked changes. You should upload this as a separate file labeled 'Manuscript'.

If you would like to make changes to your financial disclosure, please include your updated statement in your cover letter. Guidelines for resubmitting your figure files are available below the reviewer comments at the end of this letter.

If applicable, we recommend that you deposit your laboratory protocols in protocols.io to enhance the reproducibility of your results. Protocols.io assigns your protocol its own identifier (DOI) so that it can be cited independently in the future. For instructions see: <https://journals.plos.org/plosone/s/submission-guidelines#loc-laboratory-protocols>. Additionally, PLOS ONE offers an option for publishing peer-reviewed Lab Protocol articles, which describe protocols hosted on protocols.io. Read more information on sharing protocols at <https://plos.org/protocols?utm_medium=editorial-email&utm_source=authorletters&utm_campaign=protocols>.

We look forward to receiving your revised manuscript.

Kind regards,

Michael B. Steinborn, PhD
Section Editor
PLOS ONE

Response: We appreciate the positive comments from the editor. We have made revisions based on Reviewer #1's suggestions. The potential reviewers are listed in the Cover Letter, labeled as 'Suggested Reviewers'.

Journal requirements:

When submitting your revision, we need you to address these additional requirements.

1. Please ensure that your manuscript meets PLOS ONE's style requirements, including those for file naming. The PLOS ONE style templates can be found at
https://journals.plos.org/plosone/s/file?id=wjVg/PLOSOne_formatting_sample_main_body.pdf and
https://journals.plos.org/plosone/s/file?id=ba62/PLOSOne_formatting_sample_title_authors_affiliations.pdf

Response: We thank the journal staffs for the reminder about manuscript style. The format of the affiliation has been revised accordingly.

2. Thank you for stating the following financial disclosure: 
 [This study was financially sponsored through the Arts Project of 2019 National Social Science Fund of China (grant no.19BC041) and the Arts Project of 2023 National Social Science Fund of China (grant no.23ZD07). The authors thank them for financial support.].  
Please state what role the funders took in the study.  If the funders had no role, please state: "The funders had no role in study design, data collection and analysis, decision to publish, or preparation of the manuscript." 
If this statement is not correct you must amend it as needed. 
Please include this amended Role of Funder statement in your cover letter; we will change the online submission form on your behalf.

Response: We thank the journal staffs for the reminder regarding financial disclosure. We have included the Role of Funder statement. Relevant edits are listed as follows:

**On page 46:**

*“The Arts Project of 2019 National Social Science Fund of China (grant no.19BC041) had role in study design, data collection and analysis, decision to publish. The Arts Project of 2023 National Social Science Fund of China (grant no.23ZD07) had role in preparation of the manuscript. ”*

3. Please remove your figures from within your manuscript file, leaving only the individual TIFF/EPS image files, uploaded separately. These will be automatically included in the reviewers’ PDF.

Response: We thank the journal staffs for the reminder about manuscript style. There are no inserted figures in the current manuscript file.

[Note: HTML markup is below. Please do not edit.]

Reviewers' comments:

Reviewer's Responses to Questions

**Comments to the Author**

1. Is the manuscript technically sound, and do the data support the conclusions?

The manuscript must describe a technically sound piece of scientific research with data that supports the conclusions. Experiments must have been conducted rigorously, with appropriate controls, replication, and sample sizes. The conclusions must be drawn appropriately based on the data presented.

Reviewer #1: Yes

2. Has the statistical analysis been performed appropriately and rigorously?

Reviewer #1: Yes

3. Have the authors made all data underlying the findings in their manuscript fully available?

The [PLOS Data policy](http://www.plosone.org/static/policies.action#sharing) requires authors to make all data underlying the findings described in their manuscript fully available without restriction, with rare exception (please refer to the Data Availability Statement in the manuscript PDF file). The data should be provided as part of the manuscript or its supporting information, or deposited to a public repository. For example, in addition to summary statistics, the data points behind means, medians and variance measures should be available. If there are restrictions on publicly sharing data—e.g. participant privacy or use of data from a third party—those must be specified.

Reviewer #1: No

4. Is the manuscript presented in an intelligible fashion and written in standard English?

PLOS ONE does not copyedit accepted manuscripts, so the language in submitted articles must be clear, correct, and unambiguous. Any typographical or grammatical errors should be corrected at revision, so please note any specific errors here.

Reviewer #1: Yes

5. Review Comments to the Author

Please use the space provided to explain your answers to the questions above. You may also include additional comments for the author, including concerns about dual publication, research ethics, or publication ethics. (Please upload your review as an attachment if it exceeds 20,000 characters)

Reviewer #1: The presents study was conducted to test the Kuleshov effect. Kuleshov was a Russian filmmaker and theorist who famously argued that pairing shots of actors conveying a neutral expression with shots depicting different scene will induces inferences about the affective states of the person. There has been some debate on the power of the Kuleshov effect and some research suggesting his claims were overstated. The present study tested the Kuleshov effect in the context of behavioral and fMRI data. Participants viewed short film clips that depicted an actor expressing a neutral facial expression with shots intended to depict happy, fearful, or neutral context. In both experiments, emotional judgments were made regarding valence and intensity/arousal. There were clear differences in the valance judgments, but and arousal judgments (experiment 2). The fMRI data suggested that there were dissociable brain networks associated with the different conditions.

Evaluation
This study can contribute to the cognitive studies of fiction film. The experiments were well conducted. However, the literature review does not reflect contemporary thinking and research about editing practices and point of view shot sequencing (of which the Kuleshov effect is representative) and the comprehension of filmed events. Moreover, there is a burgeoning line this this research that focuses on grounding the cognitive experience of fiction film in the brain. This study will have a greater impact on the filed if the authors revise the manuscript to better reflect its relationship to the field. My review is directed at helping the authors better realize the relationship of this study to the field of the cognitive study of film and neurocinematics.

Response: We appreciate the positive comments from the reviewer. We have included contemporary thinking and research about editing practices and point-of-view shot sequencing in the Abstract, Introduction, Discussion, and Conclusion. Relevant edits are listed as follows:

**Abstract, on page 2-3:**

*“Film cognition explores the influence of cinematic elements, such as editing and film color, on viewers' perception. The Kuleshov effect, a famous example of how editing influences viewers' emotional perception, was initially proposed to support montage theory through the Kuleshov experiment. This effect, which has since been recognized as a manifestation of point-of-view (POV) editing practices, posits that the emotional interpretation of neutral facial expressions is influenced by the accompanying emotional scene in a face-scene-face sequence. ”*

*“This research also contributes to a deeper understanding of the impact of film editing on viewers' emotional perception from the contemporary POV editing practices and neurocinematic perspective, advancing the knowledge of film cognition. ”*

**Conclusion, on page 43-44:**

*“In this study, our main objective was to investigate the existence of the Kuleshov effect using authentic films within the realm of film cognition. ”*

*“Furthermore, our study contributes valuable insights to the POV structure and neurocinematics, advancing the knowledge of film cognition. ”*

Comments
1. Characterizing modern theories of the editing as montage theory is not reflective of contemporary thinking. David Bordwell’s seminal book of the structure of fiction film and his well-used textbook (Bordwell, 1985; Bordwell, Thompson, & Smith, 2019) describe editing practices as “continuity editing”. While montage was originally used to convey editing practices, it is now used to refelct a specific practice of editing shots in manner that depicts passage of time (e.g., the training scene in the movie Rocky is an example of this). As such, those most likely to read this article will not see the review as reflecting contemporary perspectives of editing practices and their impact of the cognition of fiction film.

Bordwell, D. (1985). Narration in the Fiction Film. Madison, WI: University of Wisconsin Press.
Bordwell, D., Thompson, K., and Smith, J. (2019). Film Art: An Introduction. 12th ed. McGraw-Hill.

Response: We appreciate the reviewer’s valuable suggestion. We have read Bordwell’s book and included a discussion on continuity editing in the Introduction section. The current study was conducted within the realm of film cognition, with the Kuleshov effect serving as a famous example of how film editing affects emotional perception.

The Kuleshov experiment utilized the Kuleshov sequence to support the initial montage theory, and Bordwell distinguishes between the montage sequence and the montage concept in his textbook (Bordwell & Thompson, 2008). As you suggested that the Kuleshov sequence is similar to the montage sequence (which depicts the passage of time), we have included a few sentences distinguishing these terms in the Introduction.

Bordwell’s textbook (Bordwell & Thompson 2008, CHAPTER 6, page 250):

Relevant edits are as follows:

**Introduction, on page 3:**

*“Film cognition explores the intersection of cinematic elements and viewer psychology, focusing on how features such as camera angles, editing techniques, and color influence audience perception (Bordwell, 1985; Clinton et al., 2017; Germeys & D’Ydewalle, 2007; İyilikci et al., 2023; Smith, 2012; Stadler, 2018; Yilmaz et al., 2023). Through behavioral and neuroimaging experiments (Mobbs et al., 2006; Sanz-Aznar et al., 2023; Shimamura et al., 2014), research in this field examines the impact of these filmic components on viewers' interpretation of film narratives, contributing to advancing film theory and practice (Smith, 2013).*

*Reflecting on film history, a notable example of how editing influences viewers' perception is the Kuleshov effect. Identified through an experiment conducted in the 1920s by Soviet filmmaker Lev Kuleshov (1899–1970), this study juxtaposed an image of Russian actor Ivan Mozhukin’s neutral face with various emotional contexts in a series of frames arranged in a face-scene-face sequence (Pudovkin, 1970). This sequence includes segments featuring an actor with a neutral expression, followed by an emotional scene, and concluding with a repetition of the actor's neutral expression. During this experiment, viewers were tasked with assessing the emotions portrayed by the actor's face. Despite the actor's facial expression remaining consistent, viewers frequently attributed different emotional states to the actor, deeply linked to the accompanying emotional scenes. For example, when the emotional scene depicts a dead girl lying in a cabin, viewers perceive fear in the neutral face; conversely, when the scene features an alluring woman reclining on a couch, viewers interpret happiness in the neutral face. ”*

**Introduction, on page 8:**

*“While the Kuleshov sequence is often interpreted as an example of classic montage editing, where the goal is to combine images to generate new meanings—for instance, combining a clip of Mozhukin’s neutral face with a clip of a bowl of soup may create the idea of hunger—the original Kuleshov experiment also illuminates another fundamental aspect of film editing: continuity editing. The Kuleshov sequences serve as early examples of continuity editing (Barratt et al., 2016; Bordwell & Thompson, 2008), which includes a shot-reverse-shot structure, aiming to maintain a coherent narrative space and time. Moreover, these sequences exemplify point-of-view (POV) editing practices. In the 'glance shot', a character is shown gazing toward an offscreen entity (in this instance, Mozhukin’s neutral face), whereas the 'object shot' captures the item being looked at (such as a dead girl in a cabin). The POV structure is easily comprehended by viewers because it mimics the natural human and primate tendency to follow the gaze of an intentional agent (Carroll & Russell, 1996). Thus, reexamining the Kuleshov effect, particularly how it employs the POV structure and generates new meanings from neutral faces, contributes not only to validating classic montage theory but also to revealing the efficacy of contemporary film editing practices.”*

**Distinguishing terms in Introduction, on page 8:**

*“It should be noted that the Kuleshov sequence differs from the montage sequence; the latter incorporates informative titles, newsreel footage, and music to create a quick and regular rhythm that depicts the passage of time (Bordwell & Thompson, 2008). ”*

2. The Kuleshov experiment was seminal in informing editing practices that involving pairing close-up shots of characters with other scenes to help the viewer infer what characters are thinking and feeling. A contemporary way to characterize this editing practice is point-of-view shot sequencing. The controversy over Kuleshov’s claims had everything to do with the strong argument that actors didn’t need to act, and that the editing sequence alone could convey the emotion. However, it is almost always the case that actors are asked to convey emotional expressions in point of view shot sequencies. This doesn’t change the importance of this study, but more accurately conveying the Kuleshov effect and its relationship to the practices of creating fiction film will better position this study. The authors may want to discuss the relationship of this study to contemporary studies that have either explored the impact of point of view shot sequencing or the role of closeup shots in helping viewers infer the emotional states of characters.
Bálint, K, E., Blessing, J. N., and Rooney, B. (2020). “Shot Scale Matters: the Effect of Close-up Frequency on Mental State Attribution in Film Viewers.” Poetics 83: 101480. https://doi.org/10.1016/j.poetic.2020.101480.
Clinton, J. A., Briner, S. W., Sherrill, A. M., Ackerman, T., & Magliano, J. P. (2017). “The Role of Cinematic Techniques in Understanding Character Affect.” Scientific Study of Literature, 7 (2), 177-202. https://doi.org/10.1075/ssol.16019.cli

Response: We thank the reviewer for their valuable suggestions. We have included paragraphs in the Methods and Discussion sections to discuss the relationship of this study to contemporary research that has explored the impact of point-of-view shot sequencing and the role of close-up shots in helping viewers infer the emotional states of characters. Relevant edits are listed as follows:

**Methods, on page 11:**

*“Subsequently, we combined these elements in a face-scene-face film sequence using a shot-reverse-shot structure. This setup presents the actor's face in the "shot" and the corresponding emotional scene in the "reverse shot," illustrating what the actor is supposedly observing. Thus, from a point-of-view (POV) editing perspective, the "glance shot" depicts the actor's face, while the "object shot" displays the corresponding emotional scene. ”*

**Discussion, on page 41-42:**

*“****Inferring actors’ emotions under POV structure***

*Through the POV sequence in the current experiment, we discovered that the POV structure significantly influences how viewers perceive emotions from actors. Beyond the contextual framing—where the emotional scene serves as a backdrop that shapes emotional perception (Zheng et al., 2022)—another pivotal factor is the POV structure's ability to prompt viewers to infer actors' emotions directly. This sequence in our study includes a glance shot, an object shot, and a subsequent return to the glance shot. The glance shot, a close-up, focuses the viewer on the actor's facial expression, enhancing engagement and emotional attribution. These close-up shots reveal the actor's deictic gaze, fostering social cognition and theory-of-mind in viewers (Baron-Cohen, 1997; Bloch et al., 1995; Dennett et al., 1989; Klinnert, 1984), thus enabling them to attribute nuanced mental and emotional states to the actors. As viewers hypothesize about the actors' thoughts, the ensuing object shot—aligned near the 180-degree axis—visually conveys what the actors are presumably observing from their egocentric viewpoint (Carroll & Russell, 1996), bridging the viewer's understanding of the scene's context. This method of editing not only deepens the emotional connection but also transitions the viewer from an external observer to a more immersed perspective, aligning with the actor's viewpoint.*

*Moreover, research confirms that close-up shots are pivotal in aiding viewers to gauge an actor’s emotional state effectively. For example, Balint (Bálint et al., 2020) demonstrated that increasing the frequency of close-ups significantly boosts mental state attribution and viewer engagement, a finding that supports the argument for the power of editing in enhancing emotional perception. In our research, the Kuleshov sequence incorporated two such close-up shots, which proved instrumental in helping viewers infer the actors' emotional states more accurately. Although we consistently utilized a fixed camera angle to capture the actors' neutral expressions, the influence of camera positioning in film remains a critical factor. Studies, such as those by Clinton (Clinton et al., 2017), illustrate the variations in camera angles, such as low-angle and high-angle shots, distinctly influence viewers’ emotional interpretations. The exploration of how these angles affect emotional perception will be a primary focus of our future research, aiming to further delineate the nuanced interplay between film technique and viewer emotion response. ”*

3. I do question whether the materials have ecological validity, given that actors rarely are directed to maintain neutral expressions in the context of point of view shots sequencing. I think the authors should temper their claims about this aspect of the materials given that they may not reflect the practices of commercial fiction films, despite the fact that a filmmaker was involved in their development.

Response: We appreciate the reviewer's insightful suggestion regarding the infrequent use of neutral expressions. We have tempered the claims of the materials' ecological validity in the Introduction and Limitations sections. Relevant edits are listed as follows:

**Introduction, on page 6:**

*“Therefore, the need to reexamine the Kuleshov effect using high-ecological film materials is compelling. In the current study, we opted for a neutral expression in high-ecological film materials, which rarely appears in commercial fiction films. This choice was made because it aligns with the original Kuleshov experiment, and the performance of a neutral expression can be more easily controlled. ”*

**Limitations in Discussion, on page 42:**

*“Secondly, the results from the current study may not fully reflect commercial fiction films. In such films, actors typically express a range of emotions within the POV structure (Bordwell & Thompson, 2008), and sound plays a critical role in creating an authentic film experience, potentially contributing to what might be termed the auditory Kuleshov effect (Baranowski & Hecht, 2017). Despite our intentional removal of all sound from the film sequences, in line with a previous study (Calbi et al., 2017), the potential interaction between visual and auditory Kuleshov effects and the manipulation of different facial expressions warrant further exploration. This exploration could help ascertain the extent to which our findings can be generalized to typical commercial viewing experiences. ”*

4. Uri Hasson and Gal Raz have pioneered the field of neurocinematics. Additional Gal Raz as conducted several studies grounding the affective response to film in brain networks. I would also like to draw the authors attention to the Baltic Neurocine group created by Pia Tikka (here’s a link to the 2022 conference, https://balticneurocineconf2022.sched.com/). Discussing this study in the context of the field of neuroscinematics will help the authors connect to the right readership for the article, which will help improve impact.

Response: We thank the reviewer for the valuable suggestion. We have included a paragraph describing contemporary progress in neurocinematics, including Hasson's work. Additionally, we discussed the works of Gal Raz and Pia Tikka in the Discussion. Relevant edits are listed as follows:

**Introduction, on page 6-7:**

*“In addition to reexamining the Kuleshov effect through behavioral experiments, cutting-edge neuroimaging techniques can enhance its validation by revealing its neural correlates. This approach aligns with the neurocinematic exploration of films (Hasson et al., 2008) and current study has outlined two distinct approaches to neurocinematic research (Kauttonen et al., 2015). The first employs naturalistic stimuli within fMRI environments, incorporating complex mise-en-scène and utilizing model-free analytical methods such as Independent Component Analysis (ICA) and Inter-Subject Correlation (ISC). These techniques are crucial for identifying brain activations linked to specific, annotated elements of the stimulus. For instance, Hasson used the ISC method and found that Sergio Leone’s The Good, the Bad, and the Ugly (1966) evoked similar responses across approximately 45 percent of the cortex in viewers during the movie (Hasson et al., 2008). Conversely, the second approach utilizes traditional experimental methods, notably the General Linear Model (GLM) analysis, based on event- or block-designs. In these designs, parameters for BOLD signal fluctuations indicative of brain activation are predefined (Pajula et al., 2012). When studying the current Kuleshov effect phenomena, this conventional approach is often more suitable due to the simplicity and clarity of the face-scene-face sequence, making it ideal for event-based designs. ”*

**Discussion, on page 37-38:**

*“****Comparative neurocinematic methodologies in current research***

*Film is a complex art form, and adopting neurocinematic approaches to explore its impact on viewer psychology is essential. Currently, neurocinematics predominantly utilizes two main research methodologies: Experimental Approach and Naturalistic Approach.*

*In the Experimental Approach, researchers design experiments to test specific hypotheses, such as how simple scene setups affect viewers' emotional responses (Brighter & Rader, 2019; Mobbs et al., 2006). Using the GLM method, this approach effectively investigates the mechanisms through which specific film elements impact audiences. However, the ecological validity of the materials used often does not encompass all types of films, limiting the generalizability of the findings.*

*In the Naturalistic Approach, researchers analyze large datasets to identify patterns and correlations under less controlled, more natural conditions (Hasson et al., 2008). Methods like ICA and ISC are used to manage complex scene data from films. For instance, Uri Hasson demonstrated the consistency of brain activation across viewers in response to emotional films (Hasson et al., 2008). Janne Kauttonen explored correlations between camera shots, actor movements, and brain responses in non-narrative films (Kauttonen et al., 2015). Meanwhile, Gal Raz examined changes in brain networks and dynamic brain responses to complex film scenes (Raz, Shpigelman, et al., 2016; Raz, Touroutoglou, et al., 2016). Although the complex mise-en-scène in naturalistic films limits the precise neural mapping of film elements, their research contributes to understanding the relationship between film elements and neural responses, further grounding the field of neurocinematics.*

*While these Naturalistic Approach studies have identified some common brain areas, such as the Insula-based network (Raz, Touroutoglou, et al., 2016), which align with the activation results in the current study using Experimental Approach. The complexity of the scenes often hampers the precise isolation of the independent effects of various film elements on viewer impact. Although this study employs GLM method to explore these effects, integrating the advantages of GLM method with ISC method represents a promising direction for future research. This integrated approach could offer a more comprehensive method to analyze and understand how films influence viewers' emotions and cognition through diverse film elements and brain network activities. ”*

5. The authors should consider discussing Bohn-Gettler’s Process Emotion Task (PET) Theory to help justify the study. The theory specifically discusses the relationship between affect and comprehension processes and emphasizes both valance and arousal as important dimensions. One caveat, however, is that the theory focuses on the affective experiences of the viewer/reader, not inferences about the emotions of characters.
Bohn-Gettler, Catherine M. 2019. “Getting a Grip: the PET Framework for Studying How Reader Emotions Influence Comprehension.” Discourse Processes 56 (5-6): 386–401. <https://doi.org/10.1080/0163853X.2019.1611174>.

Response: We thank the reviewer for the suggestion. After reading the PET Theory, we found that the PET framework explores how emotions affect text comprehension, such as positive emotions promoting reading and negative emotions inhibiting it. Although the PET framework is similar to our study in that both explore how emotions affect the subject’s task, the task in the current study is not reading text but rather assessing the emotion of a neutral face. The contextual framing effect in facial recognition might be more suitable to explain the current results (Wieser & Brosch, 2012). This effect suggests that when people resolve ambiguous facial emotions, they find it challenging to guess the emotion based solely on the face; instead, they utilize external cues, such as the emotional context—in this case, the emotional scenes in the current study. The emotional context affects the judgment of neutral faces in our study, leading to the Kuleshov effect bias. Relevant edits are listed as follows:

**Discussion, on page 33:**

*“According to contextual framing (Mobbs et al., 2006; Wieser & Brosch, 2012), the context-dependent bias suggests that when viewers resolve ambiguous facial emotions, they find it challenging to guess the emotion based solely on the face. Instead, they utilize external cues, such as the emotional context—in this case, the emotional scenes in the current study. The emotional context affects the judgment of neutral faces in our study, leading to the Kuleshov effect. ”*

6. I’m not sure that I understand the differences between the emotional intensity and arousal judgments for Experiments 1 and 2. Please explain and what was different in the arousal judgments that lead to them finding significant differences in Experiment 2.

Response: We thank the reviewer for the valuable suggestion. In Experiment 1, emotional intensity refers to the perceived emotional degree in the actor’s performance, whereas in Experiment 2, arousal refers to the viewer's physiological response to the neutral face. Cognitive Appraisal Theory can explain the differences in judgments between Experiment 1 and Experiment 2. Relevant edits are listed as follows:

**Discussion, on page 35-36:**

*“****Dissociation between emotional intensity rating and physiological experience of arousal rating***

*In our study, while emotional scenes influenced arousal ratings, they did not similarly affect ratings of emotional intensity. This distinction underscores the Cognitive Appraisal Theory (Richard S. Lazarus, 1984), which posits that individual appraisals of emotional stimuli significantly influence emotional experiences but do not always correspond with physiological reactions. Emotional intensity refers to the perceived emotional degree in the actor’s performance, whereas arousal measures the viewer's physiological response to the neutral face. In Experiment 1, the influence of emotional scenes on emotional intensity was not significant (F_1,2_ = 0.09, p = 0.913, as shown in Figure 3B), suggesting that participants utilized external appraisal methods and perceived similar levels of emotional intensity in the actors' performances across different emotional scenes. However, an analysis of emotional arousal in Experiment 2 revealed distinct variations across different types of emotional scenes (F_1,2_ = 4.91, p = 0.009, as shown in Figure 3D). Fearful scenes evoked the highest levels of arousal (5.27 ± 0.24), followed by happy scenes (4.94 ± 0.25), while neutral scenes registered the lowest (4.20 ± 0.25). These findings illustrate that emotional scenes (fearful and happy) can trigger heightened physiological responses compared to neutral scenes, thereby highlighting the role of cognitive appraisal in the differential emotional processing of stimuli. For example, in cognitive appraisal, fearful scenes lead to more threat perception than neutral scenes, resulting in higher arousal ratings. The divergence in findings between the two experiments reflects the distinct tasks employed: Experiment 1's focus on perceived emotional intensity and Experiment 2's measurement of physiological arousal. This demonstrates the nuanced ways viewers respond to cinematic stimuli, as cognitive appraisal may influence emotional intensity ratings, while physiological arousal may be more directly responsive to the emotional content of scenes. ”*

7. I know that the authors make materials available, but do you also make the data available? This is an important open science practice. If the data are made available, this needs to be made clearer. I usually include a data and materials access section in the methods section that specifies how to access the data

Response: We thank the reviewer for the suggestion. We have made the data available, which is detailed in the Data Availability Statement on page 45.

*“****Data availability statement***

*The experimental data presented in this article are accessible on GitHub at the following link: https://github.com/Michaelcao92/KuleshovExp. ”*

Relevant edits in the Methods section are listed as follows:

**Method, on page 35-36:**

*“The experimental materials presented in this article are accessible in the Supplementary Videos (S1-S9 Videos) and the experimental data can be accessed on GitHub at the following link: https://github.com/Michaelcao92/KuleshovExp. ”*

Joe Magliano

6. PLOS authors have the option to publish the peer review history of their article ([what does this mean?](https://journals.plos.org/plosone/s/editorial-and-peer-review-process#loc-peer-review-history)). If published, this will include your full peer review and any attached files.


If you choose “no”, your identity will remain anonymous but your review may still be made public.


**Do you want your identity to be public for this peer review?** For information about this choice, including consent withdrawal, please see our [Privacy Policy](https://www.plos.org/privacy-policy).

Reviewer #1: **Yes:** Joseph P. Magliano

Bálint, K. E., Blessing, J. N., & Rooney, B. (2020). Shot scale matters: The effect of close-up frequency on mental state attribution in film viewers. *Poetics*, *83*(July 2019), 101480. https://doi.org/10.1016/j.poetic.2020.101480

Baranowski, A. M., & Hecht, H. (2017). The Auditory Kuleshov Effect: Multisensory Integration in Movie Editing. *Perception*, *46*(5), 624–631. https://doi.org/10.1177/0301006616682754

Baron-Cohen, S. (1997). Mindblindness : an essay on autism and theory of mind. In *Cambridge University Press*. Cambridge University Press.

Barratt, D., Rédei, A. C., Innes-Ker, Å., & van de Weijer, J. (2016). Does the Kuleshov Effect Really Exist? Revisiting a Classic Film Experiment on Facial Expressions and Emotional Contexts. *Perception*, *45*(8), 847–874. https://doi.org/10.1177/0301006616638595

Bloch, M., Hirschfeld, L. A., & Gelman, S. A. (1995). Mapping the Mind: Domain Specificity in Cognition and Culture. In *Journal of the Royal Anthropological Institute*.

Bordwell, D. (1985). Narration in the Fiction Film. In *The University of Wisconsin Press*. https://doi.org/10.1525/fq.1986.40.1.04a00150

Bordwell, D., & Thompson, K. (2008). Film Art: An Introduction. In *Leonardo*.

Brighter, G., & Rader, N. (2019). Establishing shot type affects arousal and cognitive load during transitions between novel interior locations in films. *Frontiers in Human Neuroscience*, *13*(January), 1–12. https://doi.org/10.3389/fnhum.2019.00003

Calbi, M., Heimann, K., Barratt, D., Siri, F., Umiltà, M. A., & Gallese, V. (2017). How context influences our perception of emotional faces: A behavioral study on the Kuleshov effect. *Frontiers in Psychology*, *8*(OCT), 1684. https://doi.org/10.3389/fpsyg.2017.01684

Carroll, J. M., & Russell, J. A. (1996). Do Facial Expressions Signal Specific Emotions? Judging Emotion from the Face in Context. *Journal of Personality and Social Psychology*, *70*(2), 205–218. https://doi.org/10.1037/0022-3514.70.2.205

Clinton, J. A., Briner, S. W., Sherrill, A. M., Ackerman, T., & Magliano, J. P. (2017). The role of cinematic techniques in understanding character affect. *Scientific Study of Literature*, *7*(2), 177–202. https://doi.org/10.1075/ssol.16019.cli

Dennett, D. C., Dennett, D. C., Dennett, D. C., Dennett, D., & Dennett, D. (1989). The Intentional Stance. In *Philosophical Books* (Vol. 30, Issue 3).

Germeys, F., & D’Ydewalle, G. (2007). The psychology of film: Perceiving beyond the cut. *Psychological Research*, *71*(4), 458–466. https://doi.org/10.1007/s00426-005-0025-3

Hasson, U., Landesman, O., Knappmeyer, B., Vallines, I., Rubin, N., & Heeger, D. J. (2008). Neurocinematics: The Neuroscience of Film. *Projections*, *2*(1), 1–26. https://doi.org/10.3167/proj.2008.020102

İyilikci, E. A., Demirel, A., Işık, F., & İyilikci, O. (2023). How do sound and color features affect self-report emotional experience in response to film clips? *Current Psychology*. https://doi.org/10.1007/s12144-023-05127-6

Kauttonen, J., Hlushchuk, Y., & Tikka, P. (2015). Optimizing methods for linking cinematic features to fMRI data. *NeuroImage*, *110*, 136–148. https://doi.org/10.1016/j.neuroimage.2015.01.063

Klinnert, M. D. (1984). The regulation of infant behavior by maternal facial expression. *Infant Behavior and Development*, *7*(4), 447–465. https://doi.org/10.1016/S0163-6383(84)80005-3

Mobbs, D., Weiskopf, N., Lau, H. C., Featherstone, E., Dolan, R. J., & Frith, C. D. (2006). The Kuleshov Effect: the influence of contextual framing on emotional attributions. *Social Cognitive and Affective Neuroscience*, *1*(2), 95–106. https://doi.org/10.1093/scan/nsl014

Pajula, J., Kauppi, J. P., & Tohka, J. (2012). Inter-subject correlation in fMRI: Method validation against stimulus-model based analysis. *PLoS ONE*, *7*(8). https://doi.org/10.1371/journal.pone.0041196

Pudovkin, V. I. (1970). *Film Technique and Film Acting* (I. Montagu. (ed.)). New York, NY: Grove Press, Inc. https://books.google.com.br/books?id=hE98CgAAQBAJ

Raz, G., Shpigelman, L., Jacob, Y., Gonen, T., Benjamini, Y., & Hendler, T. (2016). Psychophysiological whole-brain network clustering based on connectivity dynamics analysis in naturalistic conditions. *Human Brain Mapping*, *37*(12), 4654–4672. https://doi.org/10.1002/hbm.23335

Raz, G., Touroutoglou, A., Wilson-Mendenhall, C., Gilam, G., Lin, T., Gonen, T., Jacob, Y., Atzil, S., Admon, R., Bleich-Cohen, M., Maron-Katz, A., Hendler, T., & Barrett, L. F. (2016). Functional connectivity dynamics during film viewing reveal common networks for different emotional experiences. *Cognitive, Affective and Behavioral Neuroscience*, *16*(4), 709–723. https://doi.org/10.3758/s13415-016-0425-4

Richard S. Lazarus. (1984). Stress, appraisal, and coping. In *Springer New York*.

Sanz-Aznar, J., Bruni, L. E., & Soto-Faraco, S. (2023). Cinematographic continuity edits across shot scales and camera angles: an ERP analysis. *Frontiers in Neuroscience*, *17*(July), 1–14. https://doi.org/10.3389/fnins.2023.1173704

Shimamura, A. P., Cohn-Sheehy, B. I., & Shimamura, T. A. (2014). Perceiving movement across film edits: A psychocinematic analysis. *Psychology of Aesthetics, Creativity, and the Arts*, *8*(1), 77–80. https://doi.org/10.1037/a0034595

Smith, T. J. (2012). The Attentional Theory of Cinematic Continuity. *Projections*, *6*(1), 1–27. https://doi.org/10.3167/proj.2012.060102

Smith, T. J. (2013). Watching you watch movies: Using eye tracking to inform cognitive film theory. In *Psychocinematics: Exploring Cognition at the Movies* (pp. 165–191). Oxford University Press. https://api.semanticscholar.org/CorpusID:14268626

Stadler, J. (2018). “Mind the gap”: Between movies and mind, affective neuroscience, and the philosophy of film. *Projections*, *12*(2), 86–94. https://doi.org/10.3167/proj.2018.120211

Wieser, M. J., & Brosch, T. (2012). Faces in context: A review and systematization of contextual influences on affective face processing. *Frontiers in Psychology*, *3*(NOV), 1–13. https://doi.org/10.3389/fpsyg.2012.00471

Yilmaz, M. B., Lotman, E., Karjus, A., & Tikka, P. (2023). An embodiment of the cinematographer: emotional and perceptual responses to different camera movement techniques. *Frontiers in Neuroscience*, *17*. https://doi.org/10.3389/fnins.2023.1160843

Zheng, J., Skelin, I., & Lin, J. J. (2022). Neural computations underlying contextual processing in humans. *Cell Reports*, *40*(12), 111395. https://doi.org/10.1016/j.celrep.2022.111395

[NOTE: If reviewer comments were submitted as an attachment file, they will be attached to this email and accessible via the submission site. Please log into your account, locate the manuscript record, and check for the action link "View Attachments". If this link does not appear, there are no attachment files.]

While revising your submission, please upload your figure files to the Preflight Analysis and Conversion Engine (PACE) digital diagnostic tool, <https://pacev2.apexcovantage.com/>. PACE helps ensure that figures meet PLOS requirements. To use PACE, you must first register as a user. Registration is free. Then, login and navigate to the UPLOAD tab, where you will find detailed instructions on how to use the tool. If you encounter any issues or have any questions when using PACE, please email PLOS at [figures@plos.org](mailto:figures@plos.org). Please note that Supporting Information files do not need this step.

Response: We thank the journal staffs for this reminder. We have checked the requirements for PLOS figures and updated the figures to meet these requirements.
