# Supplementary material for: Reexamining the Kuleshov effect: Behavioral and neural evidence from authentic film experiments
Source: PLoS One. 2024 Aug 5;19(8):e0308295. doi: 10.1371/journal.pone.0308295 (PMC11299807; doi:10.1371/journal.pone.0308295)
Supplement: Attachment — Submitted filename: Response to Reviewers - R2b.docx [file pone.0308295.s025.docx]

PONE-D-23-41138R1

Reexamining the Kuleshov effect: behavioral and neural evidence from authentic film experiments

PLOS ONE

Dear Dr. Wang,

Thank you for submitting your manuscript to PLOS ONE. After careful consideration, we feel that it has merit but does not fully meet PLOS ONE’s publication criteria as it currently stands. Therefore, we invite you to submit a revised version of the manuscript that addresses the points raised during the review process.

Editor comments: Fortunately, three reviewers agreed to comment on your manuscript. I ask you to revise the manuscript accordingly and to respond to each of the comments in a point-by-point manner.

Please submit your revised manuscript by Jul 22 2024 11:59PM. If you will need more time than this to complete your revisions, please reply to this message or contact the journal office at [plosone@plos.org](mailto:plosone@plos.org). When you're ready to submit your revision, log on to https://www.editorialmanager.com/pone/ and select the 'Submissions Needing Revision' folder to locate your manuscript file.

Please include the following items when submitting your revised manuscript:

- A rebuttal letter that responds to each point raised by the academic editor and reviewer(s). You should upload this letter as a separate file labeled 'Response to Reviewers'.
- A marked-up copy of your manuscript that highlights changes made to the original version. You should upload this as a separate file labeled 'Revised Manuscript with Track Changes'.
- An unmarked version of your revised paper without tracked changes. You should upload this as a separate file labeled 'Manuscript'.

If you would like to make changes to your financial disclosure, please include your updated statement in your cover letter. Guidelines for resubmitting your figure files are available below the reviewer comments at the end of this letter.

If applicable, we recommend that you deposit your laboratory protocols in protocols.io to enhance the reproducibility of your results. Protocols.io assigns your protocol its own identifier (DOI) so that it can be cited independently in the future. For instructions see: <https://journals.plos.org/plosone/s/submission-guidelines#loc-laboratory-protocols>. Additionally, PLOS ONE offers an option for publishing peer-reviewed Lab Protocol articles, which describe protocols hosted on protocols.io. Read more information on sharing protocols at <https://plos.org/protocols?utm_medium=editorial-email&utm_source=authorletters&utm_campaign=protocols>.

We look forward to receiving your revised manuscript.

Kind regards,

Michael B. Steinborn, PhD
Section Editor
PLOS ONE

Response: We appreciate the positive comments from the editor. We have made revisions based on the Reviewers’ suggestions.

[Note: HTML markup is below. Please do not edit.]

Reviewers' comments:

Reviewer's Responses to Questions

**Comments to the Author**

1. If the authors have adequately addressed your comments raised in a previous round of review and you feel that this manuscript is now acceptable for publication, you may indicate that here to bypass the “Comments to the Author” section, enter your conflict of interest statement in the “Confidential to Editor” section, and submit your "Accept" recommendation.

Reviewer #2: (No Response)

Reviewer #3: (No Response)

Reviewer #4: (No Response)

2. Is the manuscript technically sound, and do the data support the conclusions?

The manuscript must describe a technically sound piece of scientific research with data that supports the conclusions. Experiments must have been conducted rigorously, with appropriate controls, replication, and sample sizes. The conclusions must be drawn appropriately based on the data presented.

Reviewer #2: Yes

Reviewer #3: Yes

Reviewer #4: Yes

3. Has the statistical analysis been performed appropriately and rigorously?

Reviewer #2: Yes

Reviewer #3: Yes

Reviewer #4: Yes

4. Have the authors made all data underlying the findings in their manuscript fully available?

The [PLOS Data policy](http://www.plosone.org/static/policies.action#sharing) requires authors to make all data underlying the findings described in their manuscript fully available without restriction, with rare exception (please refer to the Data Availability Statement in the manuscript PDF file). The data should be provided as part of the manuscript or its supporting information, or deposited to a public repository. For example, in addition to summary statistics, the data points behind means, medians and variance measures should be available. If there are restrictions on publicly sharing data—e.g. participant privacy or use of data from a third party—those must be specified.

Reviewer #2: Yes

Reviewer #3: Yes

Reviewer #4: Yes

5. Is the manuscript presented in an intelligible fashion and written in standard English?

PLOS ONE does not copyedit accepted manuscripts, so the language in submitted articles must be clear, correct, and unambiguous. Any typographical or grammatical errors should be corrected at revision, so please note any specific errors here.

Reviewer #2: Yes

Reviewer #3: Yes

Reviewer #4: Yes

6. Review Comments to the Author

Please use the space provided to explain your answers to the questions above. You may also include additional comments for the author, including concerns about dual publication, research ethics, or publication ethics. (Please upload your review as an attachment if it exceeds 20,000 characters)

Reviewer #2: Reexamining the Kuleshov Effect: Behavioral and Neural Evidence from Authentic Film Experiments

The current study addresses an exciting and significant topic, the Kuleshov effect, by integrating behavioral and neural evidence to explore the underlying neural mechanisms. The study's rigorous experimental design and logical framework are commendable. The results are noteworthy, potentially elucidating the behavioral and neural manifestations of the Kuleshov effect. However, several critical issues need to be addressed to enhance the study's impact.

Response: We appreciate the positive comments from the reviewer. We thank the helpful suggestions from the reviewer.

Major Issues:

1. Statistical Issues:

a. For whole-brain activation, the authors used FDR correction, but the cluster size threshold was set at above 5, which is quite small. Please provide justification for the chosen cluster size criterion.

Response: We chose the default cluster size threshold in xjview because it allows us to observe a greater number of activated regions. This smaller cluster size is appropriate for our study as it helps to elucidate the underlying neural mechanisms of the Kuleshov effect. The relevant edits are listed as follows:

**Methods, on page 22:**

“This involved selecting positively activated brain regions, establishing the default cluster size threshold of five in xjview, which is deemed appropriate for our study to ensure that no critical activated regions are overlooked, necessary for understanding the neural mechanisms underlying the Kuleshov effect. Additionally, we applied false discovery rate (FDR) correction for multiple comparisons and set the significance level at p < 0.05."

b. The authors employed ANOVA followed by Fisher's Least Significant Difference for multiple comparisons. This method necessitates correction for multiple tests.

Response: We appreciate the reviewer's helpful suggestion. We applied Bonferroni correction in SPSS during the post hoc analysis. The relevant edits are listed as follows:

**Methods, on page 14:**

“Post hoc comparisons among the means were performed, employing a Bonferroni correction to account for multiple comparisons. “

**Results, on page 24-25:**

“Subsequent post hoc tests using Bonferroni correction confirmed significant differences in valence among each condition (p < 0.001), supporting the existence of the Kuleshov effect.

This correction revealed a significant main effect of emotional conditions on valence (F1.5 ,45.9 = 60.10, p < 0.001, η2 p = 0.667), with post hoc tests using Bonferroni correction revealing significant differences in valence among each condition (p < 0.001). These results reaffirmed the existence of the Kuleshov effect in an MRI scanner.

Post hoc tests using Bonferroni correction indicated significant arousal differences between fearful and neutral conditions (p < 0.001) and between happy and neutral conditions (p = 0.001). “

2. Experimental Design: On page 17, the fMRI experiment was conducted with a different presentation sequence compared to the behavioral study, which used randomization for the three conditions (happy, neutral, fearful). Could this sequential difference lead to interactions between trials? Given that emotions can persist over time, how can we ensure that the emotions induced by one trial do not influence subsequent trials?

Response: We apologize for the inadequate explanation of the randomization procedure. The trials in the happy condition were presented together, while the order of emotional conditions was randomized. Each participant experienced one of the six sequences: 'fearful-neutral-happy', 'fearful-happy-neutral', 'happy-neutral-fearful', 'happy-fearful-neutral', 'neutral-fearful-happy', or 'neutral-happy-fearful'. The frequency of each condition order was similar.

Since the ten trials in one emotional condition were presented together, emotions could persist over time before switching to another emotion. The inclusion of a jittered inter-trial interval (ITI) between trials also eliminated the possible interactions between trials. Relevant edits are listed as follows:

**Methods, on page 19:**

“Furthermore, we ensured a balanced order of emotional conditions. As there were three emotional conditions, each condition included ten trials. The ten trials for each emotional condition were presented together, while the order of emotional conditions was randomized across participants. Each participant experienced one of the six sequences: 'fearful-neutral-happy', 'fearful-happy-neutral', 'happy-neutral-fearful', 'happy-fearful-neutral', 'neutral-fearful-happy', or 'neutral-happy-fearful'. Additionally, the trials within each emotional condition were randomized. To mitigate the risk of emotional carryover effects from one trial to the next, we included a jittered ITI between each trial, allowing sufficient time for participants' emotional states to return to baseline. This approach helps ensure that the emotions induced by one trial do not influence subsequent trials."

3. The introduction and page 23 mention that the fMRI study shows that including jitters did not diminish the Kuleshov effect. Please omit this sentence as it detracts from the core objective of the study, which is to investigate the neural mechanisms of the Kuleshov effect.

Response: We appreciate the reviewer's insightful suggestion. We have omitted the sentence in the introduction and modified the sentences in the methods and results sections as follows:

**Methods, on page 20:**

“In the analysis of behavioral data, the primary objective was to examine the existence of the Kuleshov effect in the MRI scanner."

**Results, on page 25:**

“In this analysis, we sought to validate the existence of the Kuleshov effect in an MRI scanner."

4. In the fMRI analysis, three types of contrasts were used to investigate the neural basis of the Kuleshov effect. The second contrast, comparing happy or fearful conditions to the neutral condition, is similar to the third contrast, which compares Face 2 to Face 1 with the neutral condition. It would be more logical to either remove the second contrast or place it in the supplementary materials. The second contrast does not provide significant support for the current hypothesis and complicates the interpretation.

Response: We appreciate the reviewer's insightful suggestion. The second contrast has been placed in the supplementary materials. The relevant edits are listed as follows:

**Supporting information, on pages 53-55:**

“S2 Text. fMRI contrasting results. Comparison of brain activity for Face_2 between emotional and neutral conditions.

S7 Fig. Comparison of brain activity for Face_2 between emotional and neutral conditions. (A) Brain activity was obtained by subtracting Face_2 in the neutral condition from Face_2 in the fearful condition. (B) Brain activity was obtained by subtracting Face_2 in the neutral condition fromFace_2 in the happy condition. (p < 0.05, FDR-corrected, cluster size > 5 voxels).

S4 Table. fMRI Results: Face_2 in fearful or happy condition minus Face_2 in neutral condition. To probe the neural correlates of this Kuleshov effect bias, focus on how neutral faces exhibit distinct brain activation patterns when preceded by fearful or happy scenes. (p < 0.05, FDR-corrected, cluster size > 5 voxels). "

**S2_Text:**

“Supplementary results

Direct comparison of Face_2 between fearful or happy condition and neutral condition

Examining the bars corresponding to the fearful and happy conditions in Fig 3C, it becomes evident that the Kuleshov effect introduces a noticeable context-dependent bias in emotional perception. This effect alters the interpretation of neutral faces based on accompanying emotional scenes. Our subsequent analysis aimed to probe the neural correlates of this Kuleshov effect bias, focusing on how neutral faces exhibit distinct brain activation patterns when preceded by fearful or happy scenes [1].

In the fearful condition compared to the neutral condition, the contrast between Face_2 revealed significant activation in 17 clusters, involving regions such as the bilateral cerebellum, fusiform gyrus (FG), parahippocampal gyrus (PHC), angular gyrus (AG), PCC, cuneus, precuneus, precentral gyrus, and postcentral gyrus (for additional AAL atlas labels, S4 Table and S7A Fig). These regions are well-established in facial and emotional processing, supporting that the Kuleshov effect reflects a contextual modulation of face perception.

Conversely, when contrasting Face_2 in the happy condition with those in the neutral condition, only one cluster of significant activation was detected, encompassing the right cuneus, right precuneus, and right calcarine fissure and surrounding cortex (CAL) (S4 Table and S7A Fig). These regions are linked to visual processing, suggesting that the influence of happy scenes on neutral faces is comparatively weaker than fearful scenes. “

5. Interpretation of Results: The author's interpretation of the whole-brain results seems somewhat far-fetched. My view is that the whole-brain results for the fearful condition, compared to the neutral condition, show activation in sensory and motion-related regions, possibly indicating the fight-or-flight response. In contrast, the happy condition, compared to the neutral condition, shows activation in the limbic system, likely related to the reward system.

Response: We appreciate the reviewer's insightful suggestion. We have revised the interpretation of fMRI results in the discussion. The relevant edits are listed as follows:

**Discussion, on pages 36-39:**

“*Confirming the existence of the Kuleshov effect through fMRI*

The Kuleshov effect encompasses two primary interpretations. The first is that viewers perceive a new meaning, such as emotion from a neutral face, as illustrated by the first bar in Fig 3A. The second interpretation is a context-dependent bias where, compared to a neutral condition, viewers perceive significant negative or positive emotions in emotional conditions, as demonstrated by the ANOVA results of Fig 3A. To confirm the existence of the Kuleshov effect through fMRI, these interpretations were examined through contrasting analyses.

In exploring the perceived new meaning of the second neutral face (Face_2), we investigated the neural correlates associated with this interpretation. The processing of the neutral face involved contrasting the activation in Face_2 with Face_1, revealing multiple regions of activation (Fig 4, S1-S3 Tables). Specifically, as a data exploratory study, our examination of the fearful condition revealed activations in various brain regions, including the insula, cuneus, ACC, PCC, IPL, hippocampus, and OFC (Fig 4B). In contrast, the happy condition (Fig 4D) displayed distinct activation patterns compared to the fearful condition, indicating the diversity in the creation of new meaning under different emotional contexts.

To understand why these regions showed activation, we rely on the idea that engaging in movie viewing captures the complexities of real-world interactions, allowing viewers to immerse themselves in the film [36]. Thus, the Kuleshov sequence leads viewers to perceive 'more emotions' in Face_2 than in Face_1, indicating that viewers engage in the theory of mind and emotion generation. Viewers generate emotions and use the theory of mind to assume the actor experiences the same emotions. Regarding emotion, the activation of the insula—a region associated with emotion processing—is noteworthy [46]. As the activation of the ACC, PCC, and IPL was also observed, it suggests that viewers employ the theory of mind to interpret the actor’s feelings [47,48]. The rationale behind emotion perception from a neutral face could be attributed to contextual processing [40]. Zheng et al. [40], using intracranial electroencephalogram recordings, investigated context-specific modulation among the amygdala, hippocampus, and OFC. They found that the OFC modulates the hippocampus and amygdala when perceiving emotions on a neutral face. The activation of the hippocampus and OFC in this study aligns with Zheng’s findings, supporting the notion that contextual processing is a likely mechanism underlying the Kuleshov effect.

For the context-dependent bias, we initially examined activations of Face_2 in fearful and happy conditions compared to neutral conditions (S2 Text and S7 Fig), revealing distinct brain activation patterns in neutral faces when they are preceded by fearful or happy scenes. Subsequently, we conducted a comparative analysis by contrasting the activation differences between Face_2 and Face_1 in the emotional conditions with a similar contrast in the neutral condition, thereby revealing the interaction between neutral face processing and emotional conditions (Fig 5).

Several brain regions were identified as active in the contrast of Face_2 among emotional conditions (S7 Fig and S4 Table). Contrasting Face_2 between fearful and neutral conditions revealed activations in PCC, AG, and FG. The involvement of the PCC and AG, vital components of the default mode network (DMN), reinforces their role in contextual framing, particularly during the interpretation of the Kuleshov effect [49,50]. Additionally, the activation of the PCC, as noted in studies on emotional word processing [51], further supports its role in evaluating emotional meanings. The activations observed in the AG and FG during fearful conditions align with findings by Quinones Sanchez et al. [52], emphasizing heightened involvement in facial recognition and memory retrieval in fear-inducing circumstances. Contrasting Face_2 between happy and neutral conditions revealed activations in the right cuneus, precuneus, and CAL. The correlation between gray matter volume in the right precuneus and subjective happiness, as demonstrated by Sato [53], underscores the role of the precuneus in our activation results for perceiving happiness on neutral faces.

Significant brain areas were observed in the interaction between neutral face processing and emotional conditions. For the fearful contrast, the activation in sensory and motion-related areas such as the Insula, SMA, and premotor cortex may indicate a fight-or-flight response [54], as these regions are typically involved in processing environmental threats and preparing the body for rapid action, such as reacting to a fearful film sequence. For the happy contrast, activations in the limbic system areas indicate that the brain's reward pathways are engaged [55], reflecting the reward system in response to a happy film sequence that elicits pleasure.

In summary, the first interpretation of the Kuleshov effect is supported by neural results that confirm the Kuleshov sequence indeed creates new meaning for the second neutral face, supporting the montage theory that '1 + 1 > 2'. For the second interpretation, network-level brain systems were involved, generating distinct emotions after viewers watched varied film sequences. Collectively, these findings confirm the existence of the Kuleshov effect at the neural level. “

Minor Issues:

6. In the abstract, when first mentioning POV editing, please use the full term.

Response: We thank the reviewer for their suggestion. The full name of POV editing has been mentioned in the fourth line of the Abstract:

**Abstract, on page 2, line 4:**

“This effect, which has since been recognized as a manifestation of point-of-view (POV) editing practices, posits that the emotional interpretation of neutral facial expressions is influenced by the accompanying emotional scene in a face-scene-face sequence.”

7. In the whole-brain activation tables, report only the main brain region name for each cluster rather than all regions.

Response: We appreciate the reviewer’s advice regarding the reporting of tables. However, in the current study, we aim to showcase a broader range of activated regions. Therefore, we have followed the reporting style of a previous study (Smith, 2018. <https://doi.org/10.1038/s41598-018-21896-8>).

8. On page 24, in the fMRI data results section, use a results summary or conceptual results and title the subsection "Results" rather than describing the analysis process.

Response: We have renamed the section title. The relevant edits are listed as follows:

**Results, on page 26:**

“*Neutral face processing in each emotional condition*“

Reviewer #3: This is an intriguing study in which the author innovatively applies cutting-edge cognitive neuroscience technology to the field of film cognition. Utilizing rigorous experimental design based on authentic films, the study verifies the existence of the Kuleshov effect from both behavioral and neural levels. This research offers a fresh perspective for examination of the Kuleshov effect, and its findings significantly enhance our understanding of its cognitive neural mechanisms. Additionally, it holds considerable guiding significance for future integrative research. However, it might be improved notably if issues including but not limited to the following items are taken care of:

Response: We appreciate the positive comments and detailed, patient guidance from the reviewer.

Q1. The English language quality and presentation of the manuscript fall within an average range. It is recommended that professional refinement be conducted to enhance readability.
For instance, the expression "Somebody et al. [*]" is rarely used at the beginning of sentences in the main text. Moreover, the authors should vary their expressions as needed.

Response: We followed the previous Kuleshov studies (Calbi 2017, <https://www.frontiersin.org/journals/psychology/articles/10.3389/fpsyg.2017.01684/full>; Mullennix 2019, <https://journals.plos.org/plosone/article?id=10.1371/journal.pone.0224623> ) by starting with "Somebody et al. [*]" to describe the work related to the Kuleshov experiment.

Additionally, while the journal requires numerical citations within the main text, it is desirable to include the year in the main body when referencing same important studies, to allow readers to quickly grasp the timeline of the related research.

Response: We have added the publication year for important studies, allowing readers to quickly grasp the timeline of the related research.

In fact, I am not quite sure what the purpose of the penultimate paragraph in the author's introduction is, and how it relates to the context. If possible, I would appreciate it if the author could express it more clearly.

Response: The penultimate paragraph addresses Reviewer #1’s question by clarifying that the Kuleshov sequence is a type of continuity editing rather than supporting montage editing. To enhance clarity, several sentences were added to the paragraph:

**Introduction, on page 9:**

“In film history, there are two types of film editing: montage editing and continuity editing. The Kuleshov sequence encompasses both.

Although the Kuleshov sequence supports classic montage editing, it should be noted that the Kuleshov sequence differs from the montage sequence. The latter incorporates informative titles, newsreel footage, and music to create a quick and regular rhythm that depicts the passage of time [30]. “

The manuscript contains numerous verbose expressions. For example, the second paragraph in the results section of 3.1. Experiment 1 is overly detailed; the relevant information could be consolidated with post-hoc analyses. Furthermore, the caption of Figure 3 includes redundant statistical information that is already present in the main text.

Response: For the second paragraph in the results section of 3.1. Experiment 1, the detailed information is crucial as it relates to our hypothesis. If the Kuleshov effect does not exist, the valence in both the negative and happy conditions would be close to zero. The detailed valence values address this hypothesis. Including statistical information in the captions is important as it helps readers understand the paper just from the figures and captions.

The abbreviation "fMRI" first appears on line 133 (in R1_reviwer manuscript, ), yet its full name is not introduced until line 145.

Response: The abbreviation "fMRI" has been introduced earlier in the text.

Q2. The authors claim the existence of the Kuleshov effect is contentious and use it as a starting point for their research. However, in presenting the opposing views, they only reference a single, dated piece of literature (Ref 16, Prince S, 1992). Hence, it is unclear whether this controversy is confined to the period before the 21st century, or if it still persists. If the controversy mainly existed in the past and a consensus has largely been reached today, then the focus of this research might not be on addressing this dispute.

Response: Thank you for your question. The Kuleshov effect, initially identified over a century ago, necessitates validation through scientific experiments using film materials. Until the study by Prince in 1992, no rigorous scientific examination had confirmed its existence. Recently, Barratt (2016) has revisited the existence of the Kuleshov effect, albeit using static images rather than dynamic film sequences. Consequently, despite its historical recognition, the effect remains a subject of dispute as previous studies have not fully addressed it. This ongoing controversy underscores the relevance of our research focus.

Q3. The authors state that the primary goals of Experiment 1 (Exp1) and Experiment 2 (Exp2) are to verify the existence of the effect from both behavioral and neural levels. However, it appears that Exp2 also includes behavioral tests that are essentially the same as those in Exp1. The logic behind this design arrangement needs clarification from the authors.

Response: We specified the experimental condition in the first paragraph of the Methods section. Relevant edits are listed as follows:

**Methods, on page 10:**

"Subsequently, these film sequences were employed in a behavioral experiment to explore the existence of the Kuleshov effect under laptop experiment conditions. In Experiment 2, the same film sequences were utilized in an fMRI study to revalidate the Kuleshov effect within an MRI scanner environment and to investigate the neural correlates of the Kuleshov effect. Both experiments employed a within-subjects design. "

Additionally, regarding the behavioral tests, aside from the most crucial valence rating of neutral faces, the author measured Emotional Intensity in Exp1 and a different metric, Arousal, in Exp2. We hope the author can also explain the purpose behind this arrangement in the introduction.

Response: We have added a short paragraph in the Method section to explain the rationale behind the different metrics used in Experiments 1 and 2. Relevant edits are listed as follows:

**Methods, on page 11:**

"According to Cognitive Appraisal Theory [32], a dissociation exists between emotional experiences and physiological reactions. To explore this dissociation, we employed the emotional intensity scale in Experiment 1 to assess subjective emotional impacts, and the arousal scale in Experiment 2 to measure physiological responses."

Moreover, when presenting the results, although some of the results in Fig 3C&D are similar to the behavioral outcomes of Exp1, they seem misplaced in the "3.1. Experiment 1" section.

Response: We differentiated Experiment 1 and Experiment 2 with different colors and labels in the figure. Additionally, we specified the experimental conditions in the first sentence of the Results sections (3.1 and 3.2.1).

Q4. Despite the authors' claims that they utilized fMRI technology to test the existence of the Kuleshov effect (as mentioned in the last paragraph of the introduction), I believe its role is not limited to this aspect alone. Here, I would like to know whether, prior to conducting the experiments, the authors had any specific expectations for the neural results of the study based on related theories and empirical research from the perspective of the cognitive processing that associated brain regions or networks when the effect occurs, or if it was solely a data exploratory study.
Similarly, we believe that the discussion section of this study lacks depth, particularly in the discussion of the neural results. For example, on line 750, the cuneus is the result obtained from the condition subtraction. According to the principle of subtraction, the probability that this result is specific to the role of visual processing is relatively small. Conversely, it is also related to emotion, memory and verbal comprehension. The authors should interpret these brain regions or networks from the perspective of the possible cognitive processing mechanisms involved in the Kuleshov effect's production.

Response: We appreciate the reviewer's insightful suggestion. We have revised the interpretation of fMRI results in the discussion. The relevant edits are listed as follows:

**Discussion, on pages 36-39:**

“*Confirming the existence of the Kuleshov effect through fMRI*

The Kuleshov effect encompasses two primary interpretations. The first is that viewers perceive a new meaning, such as emotion from a neutral face, as illustrated by the first bar in Fig 3A. The second interpretation is a context-dependent bias where, compared to a neutral condition, viewers perceive significant negative or positive emotions in emotional conditions, as demonstrated by the ANOVA results of Fig 3A. To confirm the existence of the Kuleshov effect through fMRI, these interpretations were examined through contrasting analyses.

In exploring the perceived new meaning of the second neutral face (Face_2), we investigated the neural correlates associated with this interpretation. The processing of the neutral face involved contrasting the activation in Face_2 with Face_1, revealing multiple regions of activation (Fig 4, S1-S3 Tables). Specifically, as a data exploratory study, our examination of the fearful condition revealed activations in various brain regions, including the insula, cuneus, ACC, PCC, IPL, hippocampus, and OFC (Fig 4B). In contrast, the happy condition (Fig 4D) displayed distinct activation patterns compared to the fearful condition, indicating the diversity in the creation of new meaning under different emotional contexts.

To understand why these regions showed activation, we rely on the idea that engaging in movie viewing captures the complexities of real-world interactions, allowing viewers to immerse themselves in the film [36]. Thus, the Kuleshov sequence leads viewers to perceive 'more emotions' in Face_2 than in Face_1, indicating that viewers engage in the theory of mind and emotion generation. Viewers generate emotions and use the theory of mind to assume the actor experiences the same emotions. Regarding emotion, the activation of the insula—a region associated with emotion processing—is noteworthy [46]. As the activation of the ACC, PCC, and IPL was also observed, it suggests that viewers employ the theory of mind to interpret the actor’s feelings [47,48]. The rationale behind emotion perception from a neutral face could be attributed to contextual processing [40]. Zheng et al. [40], using intracranial electroencephalogram recordings, investigated context-specific modulation among the amygdala, hippocampus, and OFC. They found that the OFC modulates the hippocampus and amygdala when perceiving emotions on a neutral face. The activation of the hippocampus and OFC in this study aligns with Zheng’s findings, supporting the notion that contextual processing is a likely mechanism underlying the Kuleshov effect.

For the context-dependent bias, we initially examined activations of Face_2 in fearful and happy conditions compared to neutral conditions (S2 Text and S7 Fig), revealing distinct brain activation patterns in neutral faces when they are preceded by fearful or happy scenes. Subsequently, we conducted a comparative analysis by contrasting the activation differences between Face_2 and Face_1 in the emotional conditions with a similar contrast in the neutral condition, thereby revealing the interaction between neutral face processing and emotional conditions (Fig 5).

Several brain regions were identified as active in the contrast of Face_2 among emotional conditions (S7 Fig and S4 Table). Contrasting Face_2 between fearful and neutral conditions revealed activations in PCC, AG, and FG. The involvement of the PCC and AG, vital components of the default mode network (DMN), reinforces their role in contextual framing, particularly during the interpretation of the Kuleshov effect [49,50]. Additionally, the activation of the PCC, as noted in studies on emotional word processing [51], further supports its role in evaluating emotional meanings. The activations observed in the AG and FG during fearful conditions align with findings by Quinones Sanchez et al. [52], emphasizing heightened involvement in facial recognition and memory retrieval in fear-inducing circumstances. Contrasting Face_2 between happy and neutral conditions revealed activations in the right cuneus, precuneus, and CAL. The correlation between gray matter volume in the right precuneus and subjective happiness, as demonstrated by Sato [53], underscores the role of the precuneus in our activation results for perceiving happiness on neutral faces.

Significant brain areas were observed in the interaction between neutral face processing and emotional conditions. For the fearful contrast, the activation in sensory and motion-related areas such as the Insula, SMA, and premotor cortex may indicate a fight-or-flight response [54], as these regions are typically involved in processing environmental threats and preparing the body for rapid action, such as reacting to a fearful film sequence. For the happy contrast, activations in the limbic system areas indicate that the brain's reward pathways are engaged [55], reflecting the reward system in response to a happy film sequence that elicits pleasure.

In summary, the first interpretation of the Kuleshov effect is supported by neural results that confirm the Kuleshov sequence indeed creates new meaning for the second neutral face, supporting the montage theory that '1 + 1 > 2'. For the second interpretation, network-level brain systems were involved, generating distinct emotions after viewers watched varied film sequences. Collectively, these findings confirm the existence of the Kuleshov effect at the neural level. “

Q5. Regarding the method section for Exp1, the authors should directly state whether the experiment used a between-subjects design or a within-subjects design.
Additionally, I am unclear why the authors randomly divided the Neutral Face clips material into three groups beforehand. It seems that they only needed to make a random match when combining faces and emotional scenes. Of course, this is not a mistake.

Response: We appreciate the reviewer's insightful suggestion. Relevant edits are listed as follows:

**Methods, on pages 11-12:**

“Both experiments employed a within-subjects design. “

“The 30 neutral faces were randomly allocated into three groups (fearful, neutral, and happy) for the facial material rating experiment. This experiment aimed to assess the emotional expression of these neutral faces, with detailed information provided in S1 Text. Briefly, twelve participants rated the valence of the actor's neutral faces. The average valence was approximately zero (-0.02 ± 0.14), and no significant differences were observed among the three groups (F1,2 = 0.58, p = 0.569).“

Q6. In the MRI Data Acquisition section 2.2.3, there are some non-standard expressions: the matrix has no units, and the length and width of the voxel size do not correspond, with 200/64 ≈ 3mm. Another important information, the volume number, should be reported.
Regarding the preprocessing of fMRI, the authors mention that they performed Field Map correction. It should be clarified whether they collected field map data, and the relevant parameter information should be presented in section 2.2.3.
When creating the GLM model for the first-level analysis of task-based fMRI activation, were the individual head motion parameters considered?

Response: We appreciate the reviewer's insightful suggestion. Relevant edits are listed as follows:

**Methods, on pages 19-20:**

“The acquisition time for structural image data was 8 minutes. Task-related fMRI scans were performed using a T2-weighted echo planar imaging sequence (EPI/separate). The imaging parameters were as follows: TR / TE = 2000 ms / 34 ms; FA = 70°; FOV = 200 × 200 mm2; matrix size = 100 × 100; number of slices = 72; slice thickness = 2 mm; slice gap = 0 mm; voxel size = 2 × 2 × 2 mm3; volume number = 480. Fild map parameters were as follows: TR / TE1 / TE2 = 720 ms / 4.92 ms / 7.38 ms; FA = 70°; FOV = 200 × 200 mm2; matrix size = 100 × 100; number of slices = 72; slice thickness = 2 mm; slice gap = 0 mm; voxel size = 2 × 2 × 2 mm3; volume number = 1. “

Q7. When presenting the results of ANOVA on behavioral data, we hope that the authors can report the effect sizes of their analysis. Additionally, for neuroimaging analysis results, we would like to confirm if there are acturally no any significant negative activations.

Response: We reported the effect sizes in the manuscript. Relevant edits are listed as follows:

**Results, on pages 24-25:**

“To assess whether the Kuleshov effect introduced a context-dependent bias in the emotional perception of neutral faces, we conducted a repeated-measures ANOVA on the three emotional conditions (fearful, neutral, happy). Mauchly's Test of Sphericity indicated a violation of the sphericity assumption (p < 0.001), prompting the use of the Greenhouse-Geisser correction, which revealed a significant main effect of emotional conditions on valence (F1.6, 92.5 = 104.05, p < 0.001, η2 p = 0.642).

The main effect of emotional conditions on emotional intensity was not significant (F2, 57 = 0.13, p = 0.879, η2 p = 0.005, shown in Fig. 3B), suggesting that participants perceived similar emotional intensity in the actor's performances across different emotional conditions.

This correction revealed a significant main effect of emotional conditions on valence (F1.5 ,45.9 = 60.10, p < 0.001, η2 p = 0.667), with post hoc tests using Bonferroni correction revealing significant differences in valence among each condition (p < 0.001).

The results demonstrated a significant main effect of emotional conditions on arousal (F2, 29 = 9.92, p = 0.001, η2 p = 0.406). “

As the Kuleshov effect is generated during the film sequence watching, and the film watching process is not reversed, only positive activation in the current study is reasonable.

Reviewer #4: I read the present manuscript with much interest. Taking a cue from previous studies (e.g., Calbi et al., 2017; 2019) that investigated the Kuleshov effect, the authors took a further step to improve the ecological and cinematic validity of the paradigm by constructing dynamic film scenes thus making the spatial continuity between glance shots and object shots more linear and consistent. In a first experiment they basically validated stimuli and experimental paradigm, while in the second they introduced slight changes to render the paradigm suitable to an fMRI investigation. While I appreciate the methodological effort of the authors, there are several limitations and aspects to be clarified.

General comment: reading is tiring at times because there are many repetitions in the text, this lengthens an already very long text.

Response: Thank you for your highly constructive suggestions. Calbi's work has made profoundly important contributions to investigating the Kuleshov effect, and our study aims to build incrementally upon his foundational research. We deeply appreciate your detailed and patient guidance, offering numerous valuable revisions that have helped enhance the clarity and quality of our manuscript. We have made every effort to address areas of unclear description in this revision. We also hope to publish this research under your guidance.

ABSTRACT
Authors should better describe participants’ task; writing that “…participants viewed these face-scene-face film sequences and were tasked with rating the valence of neutral faces.” Is partial as the task was to evaluate also emotional intensity/arousal.

Response: Thank you for your suggestions. We have added the description. The relevant edits are listed below:

**Abstract, on page 2:**

“In Experiment 1, multiple film clips were captured under the guidance of a professional film director and seamlessly integrated into authentic film sequences. 59 participants viewed these face-scene-face film sequences and were tasked with rating the valence and emotional intensity of neutral faces.

In Experiment 2, 31 participants rated the valence and arousal of neutral faces while undergoing functional magnetic resonance imaging (fMRI). The behavioral results confirm the Kuleshov effect in the MRI scanner, while the neural data identify neural correlates that support its existence at the neural level. “

INTRODUCTION
More on the Kuleshov effect story and its narration can be found in: Kolesnikov, A. (2020). The geocultural provenance of narratives: The case of the Kuleshov effect. Film History: An International Journal, 32(2), 55-79.

Response: We have carefully read the article, *The Geocultural Provenance of Narratives: The Case of the Kuleshov Effect*, which discusses how the Kuleshov effect is interpreted differently across various cultural contexts. It explores how national and cultural histories of cinema have shaped the understanding and significance of the Kuleshov effect. Specifically, it highlights perspectives from the Soviet Union, France, and the United States, illustrating how these diverse cultural backdrops influenced the narrative and theoretical interpretation of the effect. For instance, in France, the concept was initially attributed to Pudovkin rather than Kuleshov due to the prominence of Pudovkin's works in French film studies, before being correctly reattributed to Kuleshov. This shift in attribution reflects a broader trend of cultural reinterpretation and recognition within the film academic community. Similarly, the article discusses how in America, the effect was popularized by figures like Hitchcock, who discussed it in the context of his films, further shaping its interpretation as a fundamental film editing technique. Thus, the article underscores that the Kuleshov effect, while a singular film theory, is perceived and taught differently depending on cultural and historical contexts, affecting its application and study in film academia globally.

Although we had hoped to find a direct link in the introduction, we believe Anna’s study inspires us to validate the Kuleshov effect within a Chinese population and to explore its significance in Chinese cultural context. Therefore, we have decided to reference this article more appropriately in the discussion section. We have added several sentences in the limitations section to acknowledge the global nature of film-watching. The relevant edits are listed below:

**Discussion, on pages 40-41:**

“In light of these considerations, our study aims to reexamine the existence of the Kuleshov effect within a Chinese population. Drawing on Anna Kolesnikov’s analysis of the effect’s geocultural origins [62], which validated the effect in Europe, the US, and the USSR, we explore how different cultural interpretations influence the perception and implementation of this film theory in China. This research contributes to the broader discourse by empirically demonstrating the applicability of the Kuleshov effect in Chinese culture. Our study also enriches our understanding of cross-cultural cinematic psychology.“

As for the alleged original experiment, please check whether when the contextual scene was that of a dead girl lying in a coffin the perceived emotion of the face was that of sadness.

Response: Thank you for your kind reminder. The relevant edits are listed below:

**Introduction, on page 4:**

“For example, when the emotional scene depicts a dead girl lying in a cabin, viewers perceive sadness in the neutral face; conversely, when the scene features a little girl playing with a doll, viewers interpret happiness in the neutral face [13]. “

Ref 15 is wrong, it refers to a study by P. Ekman while it should be related with the Kuleshov effect, please check and correct.

Response: Thank you for your kind reminder. Reference 15 has been removed from the manuscript as suggested. Details of the relevant edits are listed below:

**Introduction, on page 5:**

“While several studies predominantly explored the Kuleshov effect from a film-centric perspective [17,18], more recent investigations …“

As for Barratt et al. study, participants were asked to evaluate the facial expressions in terms of valence and arousal, but also choosing among nine emotional labels, so in terms of categorization. Furthermore, authors state that “Barratt discovered that participant's judgments of facial expressions were indeed influenced by the emotional stimuli, thus affirming the existence of the Kuleshov effect.”; while this was true in terms of categorization, from a dimensional point of view this happened only for sadness and desire: neutral faces paired with sad contexts were rated as the most negative and least aroused, while neutral faces paired with desire contexts were perceived as the most positive and the most aroused.

Response: Thank you for your suggestion. We have added detailed descriptions of the methods and results of Barratt's study. The relevant edits are listed below:

**Introduction, on page 5:**

“Barratt et al. (2016)[13] conducted a study employing the Kuleshov paradigm to explore how emotional contexts influence perceptions of facial stimuli. The experiment utilized neutral faces from the Karolinska Directed Emotional Faces (KDEF) picture set and paired them with online static or dynamic videos to create varied emotional stimuli. Participants were tasked with evaluating the valence and arousal of the neutral faces and additionally categorizing the expressions using nine emotional labels. While Barratt's findings confirmed that participants' judgments were influenced by these contexts, notably affirming the Kuleshov effect, this influence was predominantly evident in specific emotional categories. Particularly, neutral faces paired with sad contexts were perceived as most negative and least aroused, while those paired with contexts of desire were seen as most positive and most aroused, indicating a complex influence of contextual emotion beyond simple valence and arousal. “

Calbi et al., 2017 asked participants to evaluate the facial expression not only in terms of valence, but also of arousal and category. Please correct. And again, results clearly demonstrated the presence of a significant effect in terms of both valence and arousal but in the fear condition only. Moreover, participants tended to categorize the target person’s neutral facial expression choosing the emotion category congruent with the preceding context.

Response: Thank you for your attention to the missing details regarding arousal and category. We have addressed this oversight. The relevant edits are listed below:

**Introduction, on pages 5-6:**

“Building on this, Calbi et al. (2017)[19] sought to enhance the ecological validity of Barratt’s findings [13] by using exclusively dynamic images for reverse shots and introducing inter-trial intervals. This study further explored the contextual effects on emotion perception by asking participants to rate the valence, arousal, and emotional category of neutral faces following a Kuleshov sequence. While the results demonstrated significant effects on both valence and arousal, these effects were most pronounced in fear-inducing contexts. Moreover, participants tended to categorize the neutral facial expressions in a manner congruent with the preceding emotional context. These findings suggest that the emotional context significantly influenced the emotional judgment of neutral faces, resulting in a context-dependent bias of emotion perception. “

Regarding the passage on the different methodological approaches on neurocinematic research, I think that it should be better introduced as in its current form is somehow misleading. For instance, “In addition to reexamining the Kuleshov effect through behavioral experiments, cutting-edge neuroimaging techniques can enhance its validation by revealing its neural correlates.” Should be reframed in “In addition to reexamining the Kuleshov effect through behavioral experiments, THE USE OF cutting-edge neuroimaging techniques can enhance its validation by revealing its neural correlates.” Moreover, in the following sentence, which “current study” are the authors referring to?
Then, reading Kauttonen's introduction, I would say more that the authors describe the limitations of conventional methodology (e.g., GLM analysis) applied to fMRI studies with naturalistic stimuli (e.g., movies) in detecting brain activation related to annotated stimulus content when compared to model-free analysis methods than delineating an approach which uses naturalistic stimuli vs another which not.

Response: Thank you for your kind suggestion. We have reorganized the paragraph and removed the improper citation of Kauttonen’s study. The relevant edits are listed below:

**Introduction, on page 7:**

“In addition to reexamining the Kuleshov effect through behavioral experiments, the use of cutting-edge neuroimaging techniques can enhance its validation by revealing its neural correlates. There are two distinct approaches to neurocinematic research. The first utilizes traditional experimental methods, notably the General Linear Model (GLM) analysis, based on event- or block-designs. In these designs, parameters for BOLD signal fluctuations indicative of brain activation are predefined [26]. The second approach employs naturalistic stimuli within functional magnetic resonance imaging (fMRI) environments, incorporating complex mise-en-scène and utilizing model-free analytical methods such as Inter-Subject Correlation (ISC). These techniques are essential for identifying brain activations linked to specific, annotated elements of the stimulus. For instance, Hasson utilized the ISC method and found that Sergio Leone’s The Good, the Bad, and the Ugly (1966) evoked similar responses across approximately 45 percent of the cortex in viewers during the movie [27]. When reexamining the Kuleshov effect with fMRI, this conventional approach is often more suitable due to the simplicity and clarity of the face-scene-face sequence, making it ideal for event-based designs. “

As for Mobbs et al. fMRI study, authors should explain how the founded neural activations have been interpreted.

Response: Thank you for your suggestion. We have added detailed descriptions of how the neural activations were interpreted. The relevant edits are outlined below:

**Introduction, on pages 7-8:**

“Mobbs et al. (2006)[10] conducted an fMRI experiment that unveiled specific mechanisms involved in the Kuleshov effect, particularly regarding how emotional scenes impact neutral facial expressions. The study juxtaposed various emotional scenes with neutral facial expressions, revealing activations in distinct brain regions including the bilateral temporal pole (TP), anterior cingulate cortex (ACC), amygdala, and bilateral superior temporal sulcus (STS). The TP, which connects to structures important for processing emotional and social information such as the STS and amygdala, contributes to contextual framing by interpreting facial expressions based on prior emotional contexts. The STS plays a specific role in perceiving the dynamic aspects of faces, while the amygdala is crucial for processing socially relevant information. Additionally, the ACC is involved in schema-based appraisals, further emphasizing its role in emotional regulation. This study provided significant insights into the neural correlates of the Kuleshov effect. “

As for Calbi et al., 2019, behavioral results are partially reported as there were also valence and category evaluations.

Response: Thank you for your suggestion. We have added detailed descriptions of valence and category evaluations. The relevant edits are outlined below:

**Introduction, on page 8:**

“Similarly, Calbi et al. (2019)[28] conducted an EEG experiment that revealed specific mechanisms involved in the Kuleshov effect. In the experiment, participants were tasked with rating the valence and category of neutral faces while simultaneously undergoing EEG recording. The behavioral results replicated those of Calbi et al. (2017)[19]. “

METHODS
Regarding participants’ recruitment I have a curiosity, why such a long period to recruit them (from 2019 and 2023)?

Response: The study commenced in 2019; however, the onset of the COVID-19 pandemic in early 2020 significantly impacted global activities. In response, several lockdown measures were implemented in China to control the spread of the virus. Although these measures were effective in containing COVID-19 within China, they substantially delayed the recruitment of participants for our study. Recruitment was completed in September 2023.

How was the sample size determined (for both the experiments)?

Response: Firstly, we used G*Power software to calculate the basic sample size. We set the effect size f to 0.6, α to 0.05, power (1-β) to 0.8, and the number of groups to 3. The output for the total sample size was 30. For the behavioral experiment, we referred to previous studies; Calbi (2017) utilized 28 participants, and Mullennix (2018) utilized 44 participants. We chose 59 participants because it is larger than the output of G*Power software and greater than the sample sizes in the previous studies, which enables a more robust statistical analysis. For the fMRI experiment, we referred to the previous studies; Mobbs (2006) utilized 14 participants. We chose 31 participants because it is larger than the output of G*Power software and greater than the sample sizes in the previous studies, which ensures sufficient statistical power and resolution for detecting finer neural activities.

Relevant edits are listed as follows:

**Methods, on page 11:**

“The sample size for each experiment was determined using G*Power software and was informed by previous studies [10,19,20]. “

- EXP 1
Regarding the participants, authors should report the age range (mean and standard deviation).

Response: We have removed redundant descriptions of participants in Experiment 1 and Experiment 2, consolidating this information at the beginning of the Methods section. The mean age and standard deviation of the participants are now clearly reported there. Relevant edits are as follows:

**Methods, on page 11:**

“We recruited a cohort of 102 healthy individuals (51 females, aged 23.01 ± 3.21 years) with normal or corrected-to-normal vision from Beijing Normal University. Eligible participants, confirmed through a questionnaire, reported no history of panic disorder. Individuals majoring in film studies were deliberately excluded from participation. All provided written informed consent, approved by the ethics committee of the State Key Laboratory of Cognitive Neuroscience and Learning at Beijing Normal University (approval no. IRB B 0030 2019001). Participants were recruited between October 10, 2019, and September 16, 2023. They received monetary compensation, and the aforementioned committee ethically endorsed the experimental procedures.

We allocated 12 participants (6 females) to the materials rating experiment, 59 participants (29 females) to Experiment 1 (behavioral experiment), and 31 participants (16 females) to Experiment 2 (fMRI experiment). It was ensured that there was no participant overlap between the two experimental cohorts, thus maintaining the independence of behavioral and neural data. The sample size for each experiment was determined using G*Power software and was informed by previous studies [10,19,20]. “

As for the validation of the emotional scenes, authors should report also results of post-hoc tests.

Response: We appreciate the reviewer's insightful suggestion. Relevant edits are listed as follows:

**Methods, on page 14:**

“Post hoc tests using Bonferroni correction confirmed significant differences in valence among each group (p < 0.001). “

Did authors control for the “nature” of the content of emotional scenes across the three emotional conditions? For example, I see in Fig.1 that the happy emotional scene is depicted by a woman's face, are there similar stimuli (faces) in the other two conditions? Is there a control over the ratio between biological and non-biological ones?

Response: Thank you for your thoughtful consideration regarding the balance of emotional stimuli. We have controlled for the 'nature' of the content in the emotional scenes across the three conditions. Similar facial stimuli are present in the fearful condition (please refer to the images below). The balance between biological and non-biological elements varies: fearful (5:5), neutral (4:6), and happy (6:4). The overall ratio between biological and non-biological elements is approximately 1, ensuring consistency across conditions.

Relevant edits are listed as follows:

**Methods, on page 13:**

“The balance between biological and non-biological elements varies across conditions: fearful (5:5), neutral (4:6), and happy (6:4). The overall ratio between biological and non-biological elements is approximately 1, ensuring a balanced emotional stimulus. “

Were the valence and emotional intensity scale VAS scales? How did participants answer?

Response: Yes, both valence and emotional intensity were measured using Visual Analogue Scales, as depicted in the below images for the current study.

Valence

Emotional Intensity

Before the experiment, the experimenter explained the concepts of valence and emotional intensity. These scales were presented on paper. For valence, participants were informed that it represents the shift from displeasure (negative) to pleasure (positive) in individual perception. The scale ranged from -1 to 1: participants were asked to rate the actor's emotional valence in the film sequence by clicking the corresponding number with a mouse. For example, they would mark -1 for negative emotions, 0 for neutral, and 1 for positive emotions. Emotional intensity was defined as the perceived strength of emotion in the actor's performance, with the scale ranging from 1 to 5: 1 indicating low emotional intensity and 5 indicating high emotional intensity.

After the explanation, participants completed two practice trials to familiarize themselves with the procedure. The experimenter ensured that all participants understood the concepts of valence and emotional intensity before proceeding with the formal experiment.

Relevant edits are listed as follows:

**Methods, on page 16:**

“Both scales were Visual Analogue Scales, and participants were instructed to rate by clicking the corresponding scale value with a mouse. “

The black screen at the end the experimental trial should be called Inter-trial interval (ITI) and not ISI (which is usually between two consecutive stimuli in the same trial).

Response: The ISI has been replaced by ITI in both the main text and figures. The relevant edits are listed as follows:

**Methods, on page 16:**

“Subsequently, a 1.5-second inter-trial interval (ITI) concluded the trial, and participants seamlessly progressed to the subsequent trial, completing 30 trials (Fig 1A). “

Regarding the order of the conditions, why didn’t they follow a randomization procedure?

Response: We concur with your viewpoint; randomization is indeed a beneficial method. Experiment 1 was designed as a preliminary study, where we aimed to explore the Kuleshov effect using a fixed sequence of film clips within each condition to ensure consistency. Nevertheless, we implemented sequence balancing across conditions to mitigate order effects. In Experiment 2, which employed more rigorous experimental controls through MRI technology, we addressed the limitations of Experiment 1 by randomizing the sequence order. We observed that the results from both experiments were consistent, thereby confirming the reliability of our findings despite the varying approaches to sequence ordering.

Did authors verify ANOVA assumptions?

Response: Thank you for your attention to the statistical details. Initially, the ANOVA was performed using MATLAB's ANOVA function, which does not assess the homogeneity of variance. Following the reviewer's suggestion and further analysis, we agree that it would be appropriate to use SPSS for this task.

Considering your subsequent question, "Why did the authors this time use a repeated-measures ANOVA? Were assumptions verified?" Although the results between One-way ANOVA and repeated-measures ANOVA are not significantly different, given that our study involves a within-subject design, repeated-measures ANOVA is more suitable.

Consequently, we conducted the analysis using the repeated measures approach in SPSS software, and all statistical results have been updated. The current results now include Mauchly’s Test of Sphericity to assess the homogeneity of variance, as well as the Greenhouse-Geisser correction when the assumptions for ANOVA are not met.

Relevant edits are listed as follows:

**Methods, on page 17:**

“Subsequently, a repeated-measures analysis of variance (ANOVA) was conducted to identify statistical differences in valence among the three emotional conditions. Post hoc comparisons among the means were performed, employing a Bonferroni correction to account for multiple comparisons. The analysis was performed using IBM SPSS Statistics 24 software (https://www.ibm.com/spss?lnk=flatitem), which was similarly employed for analyzing emotional intensity. “

**Results, Exp 1, on page 24:**

“To assess whether the Kuleshov effect introduced a context-dependent bias in the emotional perception of neutral faces, we conducted a repeated-measures ANOVA on the three emotional conditions (fearful, neutral, happy). Mauchly's Test of Sphericity indicated a violation of the sphericity assumption (p < 0.001), prompting the use of the Greenhouse-Geisser correction, which revealed a significant main effect of emotional conditions on valence (F1.6, 92.5 = 104.05, p < 0.001, η2 p = 0.642). Subsequent post hoc tests using Bonferroni correction confirmed significant differences in valence among each condition (p < 0.001), supporting the existence of the Kuleshov effect.

Despite emotional scenes influencing valence ratings, no corresponding bias was observed in emotional intensity ratings. A repeated-measures ANOVA on emotional intensity showed that Mauchly's Test of Sphericity was not significant (p = 0.071), indicating that the assumption of sphericity was met. The main effect of emotional conditions on emotional intensity was not significant (F2, 57 = 0.13, p = 0.879, η2 p = 0.005, shown in Fig. 3B), suggesting that participants perceived similar emotional intensity in the actor's performances across different emotional conditions. “

How many participants did already know the Kuleshov effect?

Response: Out of the 59 participants, seven already knew the Kuleshov effect, while 52 did not. The number of participants who knew the Kuleshov effect is detailed in the caption of Figure S5, which is located at the end of the manuscript, in accordance with the PLoS One manuscript style requirements. As these are interesting results for the reader, we have also included them in the Results section. Relevant edits are listed as follows:

**Results, Exp 1, on page 24:**

“Lastly, out of the 59 participants, seven already knew the Kuleshov effect, while 52 did not. “

Which analysis/statistical model was used to assess whether prior knowledge of the Kuleshov effect or viewing frequency did impact the observation of the Kuleshov effect?

Response: We divided the participants into different groups based on their prior knowledge of the Kuleshov effect (unknown, known), and then we utilized a one-way ANOVA to assess differences in the fearful condition. Subsequent analyses were conducted in the neutral and happy conditions. Details of these analyses are as follows:

**Supporting Information, on page 54:**

“Out of the 59 participants, seven already knew about the Kuleshov effect, while 52 did not. A one-way ANOVA conducted on valence ratings across these two groups (unknown, known) revealed no significant differences between the groups, either in the fearful condition, neutral condition, or happy condition (all p > 0.1). “

“Of the 59 participants, 42 watched 0-2 films per week, 16 watched 3-5 films per week, and only one participant watched 6-8 films per week. Although there is a tendency for the middle-watching group (3-5 films per week) to have lower valence than the less-watching group (0-2 films per week), a one-way ANOVA conducted on valence ratings across the two groups (0-2 films per week, 3-5 films per week) revealed no significant differences between the groups, either in the fearful condition, neutral condition, or happy condition (all p > 0.1). “

- EXP 2
Why for the behavioral assessment in the fMRI experiment did authors use different scales (from -4 to + 4 instead of -1 +1 for valence and from 1 to 9 instead of 1-5 for arousal)?

Response: In Experiment 1, our initial aim was to validate the existence of the Kuleshov effect. The narrow range of scales used in this experiment was intended to compel participants to select a specific emotion, thereby facilitating our research objectives. However, in Experiment 2, which was conducted inside an MRI scanner, expanded scales were employed to enhance the sensitivity of our measurements. This broader range allows for a more nuanced capture of subtle variations in emotional responses, which are often less intense than those observed in typical behavioral settings due to the constraints imposed by the scanning environment.

Furthermore, from a statistical perspective, this broader range increases the variability in the data. This enhancement in variability can improve the robustness of our statistical analyses by reducing the potential for ceiling or floor effects, which might otherwise skew the interpretation of the effects being studied.

Relevant edits are listed as follows:

**Methods, on page 18:**

“The purpose of the expanded scale range in Experiment 2 was to enhance the sensitivity of our measurements and improve the robustness of our statistical analyses by reducing the potential for ceiling or floor effects. “

Why now authors employ the term arousal while in the first experiment they referred to emotional intensity? How did participants answer?

Response: We have added a detailed explanation in the Methods section to clarify the rationale behind using different terms — 'arousal' in Experiment 2 and 'emotional intensity' in Experiment 1. Participants responded using VAS scales in the MRI scanner. Details of these methodological clarifications are included as follows:

**Methods, on page 11:**

"According to Cognitive Appraisal Theory [32], a dissociation exists between emotional experiences and physiological reactions. To explore this dissociation, we employed the emotional intensity scale in Experiment 1 to assess subjective emotional impacts, and the arousal scale in Experiment 2 to measure physiological responses."

**Methods, on page 16:**

"Both scales were Visual Analogue Scales, and participants were instructed to rate by clicking the corresponding scale value with fMRI-compatible keyboards. Participants first used the two keys on the left-hand keyboard to select the value, then used the single key on the right-hand keyboard to confirm their selection."

Why did authors this time use a repeated-measure ANOVA? Were assumptions verified?

Response: Thank you for your attention to the statistical details. As noted earlier, the use of repeated-measures ANOVA is more suitable for our study due to its within-subjects design, which involves assessing the same participants across multiple conditions. Mauchly's Test of Sphericity was conducted to assess ANOVA assumptions. Relevant edits to address these points are listed as follows:

**Results, Exp 2, on page 25:**

“In this analysis, we sought to revalidate the existence of the Kuleshov effect within an MRI scanner environment. Fearful scenes elicited a negative valence rating on neutral faces (-1.48 ± 0.17), while happy scenes generated a positive valence rating on neutral faces (0.89 ± 0.16). However, neutral scenes did not induce an emotional bias in valence ratings on neutral faces (-0.16 ± 0.07), as illustrated in Fig 3C. Subsequently, a repeated-measures ANOVA was conducted. Mauchly's Test of Sphericity indicated a violation of the sphericity assumption (p = 0.005), prompting the use of the Greenhouse-Geisser correction. This correction revealed a significant main effect of emotional conditions on valence (F1.5, 45.9 = 60.10, p < 0.001, η2 p = 0.667), with post hoc tests using Bonferroni correction revealing significant differences in valence among each condition (p < 0.001). These results reaffirmed the existence of the Kuleshov effect in an MRI scanner.

Furthermore, an analysis of emotional arousal revealed that fearful scenes evoked the highest arousal levels (5.27 ± 0.24), followed by happy scenes (4.94 ± 0.25). In contrast, neutral scenes registered the lowest arousal levels (4.20 ± 0.25), as depicted in Fig 3D. A repeated-measures ANOVA was conducted, Mauchly's Test of Sphericity was not significant (p = 0.052), indicating that the assumption of sphericity was met. The results demonstrated a significant main effect of emotional conditions on arousal (F2, 29 = 9.92, p = 0.001, η2 p = 0.406). Post hoc tests using Bonferroni correction indicated significant arousal differences between fearful and neutral conditions (p < 0.001) and between happy and neutral conditions (p = 0.001). These findings suggest that emotional scenes triggered higher emotional responses than neutral scenes. Importantly, none of the participants reported prior knowledge of the Kuleshov effect. “

ANOVA statistical results of Exp 1 and 2 should be accompanied by the effect size.

Response: We reported the effect sizes in the manuscript. Relevant edits are listed as follows:

**Results, on page 24:**

“To assess whether the Kuleshov effect introduced a context-dependent bias in the emotional perception of neutral faces, we conducted a repeated-measures ANOVA on the three emotional conditions (fearful, neutral, happy). Mauchly's Test of Sphericity indicated a violation of the sphericity assumption (p < 0.001), prompting the use of the Greenhouse-Geisser correction, which revealed a significant main effect of emotional conditions on valence (F1.6, 92.5 = 104.05, p < 0.001, η2 p = 0.642).

The main effect of emotional conditions on emotional intensity was not significant (F2, 57 = 0.13, p = 0.879, η2 p = 0.005, shown in Fig. 3B), suggesting that participants perceived similar emotional intensity in the actor's performances across different emotional conditions.

This correction revealed a significant main effect of emotional conditions on valence (F1.5, 45.9 = 60.10, p < 0.001, η2 p = 0.667), with post hoc tests using Bonferroni correction revealing significant differences in valence among each condition (p < 0.001).

The results demonstrated a significant main effect of emotional conditions on arousal (F2, 29 = 9.92, p = 0.001, η2 p = 0.406). “

DISCUSSION
In general, I would suggest authors to rewrite the discussion to shorten it and make it smoother. The discussion opens, in fact, with a section (which we might call “general discussion”) that seems to exhaust all its relevant points in a superficial way only to find that it is followed by a series of in-depth sections. The authors may think not anticipating too much or stating that this is an initial general discussion to be followed by specific paragraphs devoted to each topic.

Response: Thank you for your constructive suggestions. We have shortened the general discussion and separated the discussion of behavioral results from Experiment 1 and Experiment 2 into a section, titled “Confirming the Existence of the Kuleshov Effect Through Behavioral Experiments”. Relevant edits are as follows:

**Discussion, on page 31:**

"Lev Kuleshov conducted the seminal Kuleshov experiment nearly a century ago, which featured a face-scene-face sequence [12]. The experiment provided foundational support for the original montage theory and was a pioneering effort to explore the impact of film elements on viewers' perception within the realm of film cognition. The Kuleshov structure also utilized contemporary POV editing techniques. From both classical theoretical evidence and contemporary film cognition perspectives, revisiting the Kuleshov effect not only reexamines its existence but also highlights the efficacy of contemporary film editing practices. This study aims to confirm the existence of the Kuleshov effect through behavioral and fMRI experiments.

*Confirming the existence of the Kuleshov effect through behavioral experiments*

While the Kuleshov effect stands as a cornerstone in film studies, the early evidence from the original Kuleshov experiment is often considered ambiguous due to its limited experimental controls and the subjective assessments of the viewers' responses. It lacks clarity [12], leaving the existence of the Kuleshov effect an open question. Over the past three decades, despite replicated experiments suggesting the detection of the Kuleshov effect [16,17,19,20], many relied on inauthentic film materials, such as static images, which may not accurately capture the dynamic nature of actual film viewing. Consequently, their findings may not generalize to the existence of the Kuleshov effect in an authentic film scenario.

In the current study, we utilized authentic film materials (S1 Fig) and conducted two experiments using the face-scene-face Kuleshov sequence (Fig 1B and Fig 2). Although a rating experiment established a baseline for neutral faces, participants were asked to rate the valence of the neutral face after watching the face-scene-face sequence, revealing that the emotional scene indeed affects the perception of emotion from a neutral face, whether viewed on a laptop (Fig 3A) or in an MRI scanner (Fig 3C). The ANOVA results showed a significant difference in valence between emotional and neutral conditions, suggesting that a viewer's interpretation of an actor's expression is significantly influenced by the emotional scene, leading to a context-dependent bias in emotional perception. According to contextual framing [10,35], this context-dependent bias indicates that when viewers resolve ambiguous facial emotions, they rely on external cues, such as the emotional context—in this case, the emotional scenes in the current study. The emotional context influences the judgment of neutral faces in our study, supporting the Kuleshov effect.

Notably, the Kuleshov sequence triggered higher arousal in the fearful condition compared with the neutral condition, consistent with prior research [21]. Additionally, arousal levels in the happy condition were also higher than in the neutral condition. These observations suggest that the emotional conditions initiate a context-dependent bias and intensify viewers' emotional experiences. Effective control of the influence of prior knowledge about the Kuleshov effect ensures the reliability of our experimental results, as depicted in Supplementary Figure (S5 Fig). In line with previous studies [19,21], the behavioral results from Experiments 1 and 2 collectively support the existence of the Kuleshov effect in an authentic film scenario."

In addition, we reviewed the logic of the paragraphs and reorganized the sections within the discussion. We grouped the behavioral sections together and the neuroimaging sections together. The revised order of sections is now as follows:

"*Confirming the Existence of the Kuleshov Effect through Behavioral Experiments*

*Enhancing the Ecological Validity of the Kuleshov Effect with Authentic Films*

*Dissociation between Emotional Intensity Rating and Physiological Experience of Arousal*

*Inferring Actors’ Emotions under Point-of-View (POV) Structure*

*Confirming the Existence of the Kuleshov Effect through fMRI*

*Comparative Neurocinematic Methodologies in Current Research*

*Limitations and Future Directions*"

The authors might consider discussing why in Exp1 they found significant results only on the valence assessment, while the emotional intensity results were not significant. I think it is very interesting, especially because in experiment 2 they got significant differences in arousal as well. Regarding this, why authors state only that “the Kuleshov sequence triggered higher arousal in the fearful condition compared with the neutral condition” if this was true also for happy condition?

Response: Yes, it was true for the happy condition. The post-hoc comparison between happy and neutral on the arousal scale shows the results. Relevant edits are listed as follows:

**Methods, on page 32:**

"Additionally, arousal levels in the happy condition were also higher than in the neutral condition. These observations suggest that the emotional conditions initiate a context-dependent bias and intensify viewers' emotional experiences. "

I see that they dedicate a section on the difference between emotional intensity and arousal in another section, so maybe they can consider reframing or reorganizing the text to allow for a smoother reading (see my previous general point).

Response: We have added a short paragraph in the Method section to explain the rationale for using emotional intensity in Experiment 1 and arousal in Experiment 2. Relevant edits are listed as follows:

**Methods, on page 11:**

"According to Cognitive Appraisal Theory [31], a dissociation exists between emotional experiences and physiological reactions. To explore this dissociation, we employed the emotional intensity scale in Experiment 1 to assess the subjective perceived emotional degree in the actor’s performance, and the arousal scale in Experiment 2 to measure physiological responses to the neutral face."

Moreover, which question was exactly asked to participants to evaluate emotional intensity/arousal? How did authors explain the meaning of both to participants? (this

Response: Firstly, we introduced the concepts of emotional intensity and arousal to the participants. Emotional intensity is defined as the perceived emotional degree in the actor's performance, indicating how strongly the emotions are expressed. Arousal, conversely, measures the viewer's physiological response to the neutral face, reflecting the level of physiological alertness triggered by the performance.

To aid their understanding, we provided participants with a printed Visual Analogue Scale (VAS) before the practice session. Participants were instructed on how to mark their ratings for both emotional intensity and arousal as follows:

For emotional intensity: "Please rate the emotional intensity of the actor's face, where 1 represents 'very weak' and 5 represents 'very intense'."

For arousal: "Please rate the arousal of the actor's face, where 1 represents the lowest level of arousal, 5 represents a neutral level, and 9 represents the highest level of arousal."

During the formal experiment, participants watched the film sequence and independently rated emotional intensity or arousal using the VAS scales, which were provided in Chinese.

The section “Enhancing ecological validity of the Kuleshov effect with authentic films” does not add much relevant info but seems more like a repetition of many things explained already in the methods, can be shortened considerably to get straight to the point.

Response: Thank you for your constructive suggestions. We have shortened this section. Relevant edits are listed as follows:

**Discussion, on page 33:**

"*Enhancing ecological validity of the Kuleshov effect with authentic films*

As the Kuleshov effect is a film editing phenomenon, validating this effect should ideally involve continuous films, rather than isolated images or clips. Earlier investigations [13,19,21] relied on facial expressions from the KDEF picture set and utilized zoom-in techniques to simulate dynamic faces from static images. These efforts faced challenges in achieving realism, including subtle facial tremors induced by individuals remaining still, the natural occurrence of eye blinking during observations, and the static background [22,23,37], making the simulation of static images as real-life films difficult. Moreover, earlier investigations selected face and emotional scenes separately rather than matching them as an integrated film [13,21]. Since the films closely mirror reality [38,39], adherence to continuity-editing rules aid viewers in perceiving the film as an emulation of the real world [7,40,41]. Consequently, using isolated neutral faces and emotional scenes separately, without considering the coherence of the backgrounds, led to spatial discontinuities.

To address these challenges, we employed a meticulous approach to capture neutral facial expressions against a blue background and filmed emotional scenes, along with their surrounding environment, in high resolution. Subsequently, we replaced the background with surrounding images or videos (S1 Fig and S1-S9 Videos), aiming for a seamless integration of neutral faces into the film's context [41]. These rigorous methodologies guarantee a thorough reexamination of the Kuleshov effect in an authentic film scenario, eliminating potential confounding factors arising from neutral faces in the observation of the Kuleshov effect bias. "

I do not entirely agree with this statement “In contrast to studies that employed neutral faces from the KDEF picture set without assessing distinct groups, our approach revealed no significant differences among faces in different conditions (F1,2 = 1.23, p = 0.305).” because in those cases stimuli were selected from an already validated dataset while this time authors built their own stimuli and they obviously had to validate them. Moreover, authors should check for the reported statistical values as they are different from those enlisted in the methods (page 13 line 277 of the revised manuscript with track changes).

Response: We have removed the comparison between the materials experiment and the KDEF picture set. The relevant edits are as follows:

**Discussion, on page 33:**

"To address these challenges, we employed a meticulous approach to capture neutral facial expressions against a blue background and filmed emotional scenes, along with their surrounding environment, in high resolution. Subsequently, we replaced the background with surrounding images or videos (S1 Fig and S1-S9 Videos), aiming for a seamless integration of neutral faces into the film's context [41]. These rigorous methodologies guarantee a thorough reexamination of the Kuleshov effect in an authentic film scenario, eliminating potential confounding factors arising from neutral faces in the observation of the Kuleshov effect bias. "

Why did authors separate the discussion of neural correlats in two sections of the discussion?

Response: We appreciate your insightful suggestion. The Kuleshov effect encompasses two primary interpretations. The first is that viewers perceive new meaning, such as emotion, from a neutral face. The second interpretation is a context-dependent bias, where, compared to a neutral condition, viewers perceive significant negative or positive emotions in emotional conditions. To confirm the existence of the Kuleshov effect through fMRI, these interpretations were examined through contrasting analyses.

In the previous version of the manuscript, we felt one section dedicated to confirming the existence of the Kuleshov effect was too lengthy; therefore, we split it into two separate sections. However, your insightful question prompted a reevaluation of the logic. Consequently, I have restated the two interpretations of the Kuleshov effect and combined the two sections into one. The relevant edits are listed as follows:

**Discussion, on pages 36-39:**

"*Confirming the existence of the Kuleshov effect through fMRI*

The Kuleshov effect encompasses two primary interpretations. The first is that viewers perceive a new meaning, such as emotion from a neutral face, as illustrated by the first bar in Fig 3A. The second interpretation is a context-dependent bias where, compared to a neutral condition, viewers perceive significant negative or positive emotions in emotional conditions, as demonstrated by the ANOVA results of Fig 3A. To confirm the existence of the Kuleshov effect through fMRI, these interpretations were examined through contrasting analyses.

In exploring the perceived new meaning of the second neutral face (Face_2), we investigated the neural correlates associated with this interpretation. The processing of the neutral face involved contrasting the activation in Face_2 with Face_1, revealing multiple regions of activation (Fig 4, S1-S3 Tables). Specifically, as a data exploratory study, our examination of the fearful condition revealed activations in various brain regions, including the insula, cuneus, ACC, PCC, IPL, hippocampus, and OFC (Fig 4B). In contrast, the happy condition (Fig 4D) displayed distinct activation patterns compared to the fearful condition, indicating the diversity in creation of new meaning under different emotional contexts.

To understand why these regions showed activation, we rely on the idea that engaging in movie viewing captures the complexities of real-world interactions, allowing viewers to immerse themselves in the film [36]. Thus, the Kuleshov sequence leads viewers to perceive 'more emotions' in Face_2 than in Face_1, indicating that viewers engage in the theory of mind and emotion generation. Viewers generate emotions and use the theory of mind to assume the actor experiences the same emotions. Regarding emotion, the activation of the insula—a region associated with emotion processing—is noteworthy [46]. As the activation of the ACC, PCC, and IPL was also observed, it suggests that viewers employ the theory of mind to interpret the actor’s feelings [47,48]. The rationale behind emotion perception from a neutral face could be attributed to contextual processing [40]. Zheng et al. [40], using intracranial electroencephalogram recordings, investigated context-specific modulation among the amygdala, hippocampus, and OFC. They found that the OFC modulates the hippocampus and amygdala when perceiving emotions on a neutral face. The activation of the hippocampus and OFC in this study aligns with Zheng’s findings, supporting the notion that contextual processing is a likely mechanism underlying the Kuleshov effect.

For the context-dependent bias, we initially examined activations of Face_2 in fearful and happy conditions compared to neutral conditions (S2 Text and S7 Fig), revealing distinct brain activation patterns in neutral faces when they are preceded by fearful or happy scenes. Subsequently, we conducted a comparative analysis by contrasting the activation differences between Face_2 and Face_1 in the emotional conditions with a similar contrast in the neutral condition, thereby revealing the interaction between neutral face processing and emotional conditions (Fig 5).

Several brain regions were identified as active in the contrast of Face_2 among emotional conditions (S7 Fig and S4 Table). Contrasting Face_2 between fearful and neutral conditions revealed activations in PCC, AG and FG. The involvement of the PCC and AG, vital components of the default mode network (DMN), reinforces their role in contextual framing, particularly during the interpretation of the Kuleshov effect [49,50]. Additionally, the activation of the PCC, as noted in studies on emotional word processing [51], further supports its role in evaluating emotional meanings. The activations observed in the AG and FG during fearful conditions align with findings by Quinones Sanchez et al. [52], emphasizing heightened involvement in facial recognition and memory retrieval in fear-inducing circumstances. Contrasting Face_2 between happy and neutral conditions revealed activations in the right cuneus, precuneus, and CAL. The correlation between gray matter volume in the right precuneus and subjective happiness, as demonstrated by Sato [53], underscores the role of the precuneus in our activation results for perceiving happiness on neutral faces.

Significant brain areas were observed in the interaction between neutral face processing and emotional conditions. For the fearful contrast, the activation in sensory and motion-related areas such as the Insula, SMA and premotor cortex may indicate a fight-or-flight response [54], as these regions are typically involved in processing environmental threats and preparing the body for rapid action, such as reacting to a fearful film sequence. For the happy contrast, activations in the limbic system areas indicate that the brain's reward pathways are engaged [55], reflecting the reward system in response to a happy film sequence that elicits pleasure.

In summary, the first interpretation of the Kuleshov effect is supported by neural results that confirm the Kuleshov sequence indeed creates new meaning for the second neutral face, supporting the montage theory that '1 + 1 > 2'. For the second interpretation, network-level brain systems were involved, generating distinct emotions after viewers watched varied film sequences. Collectively, these findings confirm the existence of the Kuleshov effect at the neural level. "

Also, we made changes in the Introduction and the Methods. The relevant edits are as follows:

**Introduction, on page 10:**

"Initially, our goal was to observe how viewers perceive new meaning within the Kuleshov effect by contrasting activation between faces. Subsequently, we examined the context-dependent bias of the Kuleshov effect, investigating how brain activity elicited by faces was altered by emotional scenes. "

**Methods, on pages 21-22:**

"We then examined the interactions between neutral face processing and emotional conditions by comparing the (Face_2 – Face_1) contrast in emotional conditions with the corresponding contrast in the neutral condition. "

MINOR:
line 268 (revised manuscript with track changes): and.
Line 502 (revised manuscript with track changes): not within but among

Response: We appreciate the reviewer's insightful suggestion. Relevant edits are listed as follows:

**Methods, on page 12:**

“Throughout the recording, actors kept their facial muscles relaxed, minimizing movements to blinking only, and we recorded 51 face clips from various actors, each cut into a 2-second clip.”

**Results, on page 24:**

“Subsequent post hoc tests using Bonferroni correction confirmed significant differences in valence among each condition (p < 0.001), supporting the existence of the Kuleshov effect.

7. PLOS authors have the option to publish the peer review history of their article ([what does this mean?](https://journals.plos.org/plosone/s/editorial-and-peer-review-process#loc-peer-review-history)). If published, this will include your full peer review and any attached files.


If you choose “no”, your identity will remain anonymous but your review may still be made public.


**Do you want your identity to be public for this peer review?** For information about this choice, including consent withdrawal, please see our [Privacy Policy](https://www.plos.org/privacy-policy).

Reviewer #2: **Yes:** Jingyuan Ren

Reviewer #3: No

Reviewer #4: No

[NOTE: If reviewer comments were submitted as an attachment file, they will be attached to this email and accessible via the submission site. Please log into your account, locate the manuscript record, and check for the action link "View Attachments". If this link does not appear, there are no attachment files.]

While revising your submission, please upload your figure files to the Preflight Analysis and Conversion Engine (PACE) digital diagnostic tool, <https://pacev2.apexcovantage.com/>. PACE helps ensure that figures meet PLOS requirements. To use PACE, you must first register as a user. Registration is free. Then, login and navigate to the UPLOAD tab, where you will find detailed instructions on how to use the tool. If you encounter any issues or have any questions when using PACE, please email PLOS at [figures@plos.org](mailto:figures@plos.org). Please note that Supporting Information files do not need this step.
